# Five long non-coding RNAs establish a prognostic nomogram and construct a competing endogenous RNA network in the progression of non-small cell lung cancer

**DOI:** 10.1186/s12885-021-08207-7

**Published:** 2021-04-23

**Authors:** Yong Yu, Kaiming Ren

**Affiliations:** 1 Shengjing Hospital of China Medical University, Shenyang, 110004 Liaoning China; 2 Department of Thoracic Surgery, Shengjing Hospital of China Medical University, Shenyang, 110004 Liaoning China

**Keywords:** NSCLC, Long non-coding RNA, Nomogram, ceRNA, Overall survival

## Abstract

**Background:**

Accumulating evidence has revealed that long non-coding RNAs (lncRNAs) play vital roles in the progression of non-small cell lung cancer (NSCLC). But the relationship between lncRNAs and survival outcome of NSCLC remains to be explored. Therefore, we attempt to figure out their survival roles and molecular connection in NSCLC.

**Methods:**

By analyzing the transcriptome profiling of NSCLC from TCGA databases, we divided patients into three groups, and identified differentially expressed lncRNAs (DELs) of each group. Next, we explored the prognostic roles of common DELs by univariate and multivariate Cox analysis, Lasson, and Kaplan-Meier analysis. Additionally, we assessed and compared the prognostic accuracy of 5 lncRNAs through ROC curves and AUC values. Ultimately, we detected their potential function by enrichment analysis and molecular connection through establishing a competing endogenous RNA (ceRNA) network.

**Results:**

One hundred ninety-seven common DELs were spotted. And we successfully screened out 5 lncRNAs related to the patient’s survival, including LINC01833, AC112206.2, FAM83A-AS1, BANCR, and HOTAIR. Combing with age and AJCC stage, we constructed a nomogram that prognostic prediction was superior to the traditional parameters. Furthermore, 275 qualified mRNAs related to 5 lncRNAs were spotted. Functional analysis indicates that these lncRNAs act key roles in the progression of NSCLC, such as P53 and cell cycle signaling pathway. And ceRNA network also suggests that these lncRNAs are tightly connected with tumor progression.

**Conclusions:**

A nomogram and ceRNA network based on 5 lncRNAs indicate that there can effectively predict the overall survival of NSCLC and potentially serve as a therapeutic guide for NSCLC.

**Supplementary Information:**

The online version contains supplementary material available at 10.1186/s12885-021-08207-7.

## Background

Non-small cell lung cancer (NSCLC) is one of the most common and deadly cancers in the world. Despite advances in treatments, only 19% of patients with NSCLC survival for more than 5 years [1, 2]. What’s worse, due to lacking specific symptoms in the early stage, most patients seek treatment at an advanced stage, which misses the best timing for a radical operation [3]. Therefore, it is urgent to establish a prognostic risk-score model for NSCLC patients, thereby providing a therapeutic guide for NSCLC.

Long non-coding RNAs (lncRNAs) refers to non-protein-coding transcripts over 200 nucleotides in length [4]. Although lncRNAs do not directly encode RNA, it can regulate protein expression at various stages of transcription [5, 6]. Based on the ceRNA hypothesis, lncRNAs, messenger RNAs, and pseudogenes can “talk” to each other using miRNA response elements (MREs) and assemble as a ceRNA network [7]. In this network, lncRNA act as “sponges” to absorb and bind miRNA, thereby weakening their binding ability to mRNA and regulating gene expression. Accumulating evidence has backed that lncRNAs were involved in the ceRNA network of many types of cancers, including pancreatic cancer, gastric cancer, as well as NSCLC [8–10]. Notably, LncRNAs have great advantages as biomarkers because they are stable, highly tissue-specific, and easy to detect in body fluids. Besides, some lncRNAs have been recognized as a novel biomarker, for instance, BANCR in gastric carcinoma, HOTAIR in colorectal carcinoma, and MALAT1 in lung cancer [11–13]. Therefore, it is needful to detect the prognostic relationship between lncRNAs and NSCLC.

In this study, we performed a large sample analysis to find out survival-related lncRNAs and validated it using univariate, Lasson, and multivariate Cox proportional hazards regression (CPHR). Moreover, combing with age and AJCC stage, we constructed a nomogram based on lncRNAs, which performed better prognostic prediction than clinical factors, and we successfully assessed its efficiency in LUAD and LUSC groups. Functional analysis indicated that these lncRNAs also act important roles in the progression of NSCLC, for example, the P53 signaling pathway and cell cycle pathway. Next, we successfully constructed a ceRNA network related to 5 lncRNAs. Those results indicated that those lncRNAs not only effectively predict the prognosis of NSCLC patients but also take part in the progression of NSCLC and potentially serve as a therapeutic target.

## Methods

### Data selection and process

As is well know that the majority of NSCLC is composed of lung squamous carcinoma (LUSC) and lung adenocarcinoma (LUAD). So we obtained the raw counts of transcriptome profiling and clinical data from TCGA-LUAD including 535 cancer samples and 59 non-tumor tissues, and data from TCGA-LUSC including 502 cancer samples and 49 non-tumor tissues. We obtained the RNA-Seq data for lncRNA and mRNA analysis and downloaded the miRNA-Seq data for miRNA analysis.

Since there was only one patient in the TCGA-MESO database, we included TCGA_LUAD and TCGA_LUSC in the NSCLC group with 1145 patients. Then we divided those data into three groups including the NSCLC group, LUAD group, and LUSC group. All the raw count data of lncRNAs, miRNAs, and mRNAs were obtained from The Cancer Genome Atlas (TCGA) database and were annotated through the Ensemble database (Homo_sapiens.GRCh38.99) [14, 15]. Stepwise, all the data were log2 (x + 1) transformed and normalized using the “LIMMA” package [16]. The “LIMMA” package is a widely used tool for calculating gene differential expression [17, 18]. This calculation process is performed automatically by R software. Additionally, to avoid bias caused by low expression genes, we removed differential genes with low average expression. Then, we screened out differentially expressed miRNAs (DEMIs) in three groups by the “LIMMA” package with the criteria of |log2FC| > 1, average expression > 1, and adjust *P*-value < 0.05, and identified differentially expressed lncRNAs (DELs) and differentially expressed mRNAs (DEMs) in three groups with the criteria of |log2FC| > 2, average expression > 2, and adjust *P*-value < 0.05, respectively. Finally, we selected the DELs, DEMs and DEMIs that both expressed in three groups for further analysis to reduce the bias caused by a single database.

### Survival analysis

Additionally, we removed samples without survival time or survival time less than 7 days to improve the reliability of our study. We first estimated the association between overall survival (OS) and clinical parameters through univariate and multivariate CPHR analysis. Furthermore, we evaluated the relationship between survival time and common DELs expression through Kaplan Meier analysis and univariate CPHR method. Only DELs that their *P*-value was lower than 0.05 and their expression consistent with prognosis were regarded as candidate survival-related lncRNAs. Combing with clinical risk factors, we performed Lasson Cox regression analysis to obtain the best fitting variables. After selecting the best-fit of OS-related variables by the calculation mentioned above, we further verified their prognostic value through multivariate CPHR analysis. And only variables that *P*-value was lower than 0.05 in univariate and multivariate CPHR calculations were selected as qualified lncRNAs and were chose for the next analysis.

### Constructing a risk score formula and nomogram model

Combining with clinical risk variables and qualified lncRNAs, we performed univariate and multivariate CPHR analysis to identify OS-related biomarkers in 970 patients with NSCLC. Also, we calculated the prognostic risk score of each patient through multivariate CPHR analysis according to the formula as follows: risk score = X1α1 + X2α2 + X3α3 + ... + Xnαn. And patients in the NSCLC group were divided into high-risk groups and low-risk groups based on the median value of risk score. C-indexes were performed to assess the predictive performances of our risk score formula. Next, we evaluated the prognostic differences in high-risk and low-risk groups by Kaplan Meier analysis and T-test. We assessed the prediction performance of the risk formula through ROC curves at 3 and 5 years and computed their AUC values in three groups. Moreover, we established a nomogram to vividly depict the predictive relationship among clinical factors, lncRNAs, and OS. Calibration curves of 3 and 5 years were calculated to assess the reliability of OS prediction between predicted performance and actual ability. All the analyses mentioned above were conducted in NSCLC, LUAD, and LUSC groups, respectively.

### Functional enrichment analysis

We performed the Gene Ontology (GO) terms and Kyoto Encyclopedia of Gene and Genomes (KEGG) pathways enrichment analysis to elucidate the potential functions of lncRNAs in the nomogram. The common DEMs in the three groups were first identified. Next, the correlation coefficient of each lncRNA with common DEMs was calculated, respectively. To obtain an accurate result, we only selected DEMs with a correlation coefficient greater than 0.2 for further enrichment analysis. Then, we performed the GO and KEGG analysis of lncRNA-related DEMs via the DAVID database, and the Enrichr database [19, 20]. And we depicted the top 10 enriched GO terms and KEGG pathways through a bar plot with the criteria of adjusting *P*-value < 0.05.

### Establishing a ceRNA network

To explore the potential interaction between 5 lincRNAs and miRNAs, the LncBase database that provided miRNAs and lncRNAs interactions according to MREs sites was applied to predict the downstream miRNA of 5 lncRNAs with the criteria of Prediction score > 0.8 [21]. Only miRNAs expressed on the NSCLC group, LUAD group, LUSC group, and LncBase database were chosen as qualified miRNAs. Additionally, the miRDB database and the miRTarBase database are a widely-used tool for miRNA target prediction that were employed to find out potential mRNAs binding to qualified miRNAs [22, 23]. Only miRNAs that validated in miRDB database, miRTarBase database, and DEMs were considered as candidate mRNAs. Finally, to vividly display the interaction of 5 lncRNAs with qualified miRNAs and candidate mRNAs, we constructed a ceRNA network by Cytoscape software (Version 3.7.2) [24].

### Statistical analysis

We performed all the statistical analyses mentioned above using R software (version 3.6.1). Briefly, OS was analyzed using the Kaplan–Meier test, and the log-rank T-test was applied to calculate the statistical significance. Univariate and multivariate CPHR analyses were conducted through “Survival” packages (Version 3.2–7) [25]. Lasson CPHR was performed using “Survival” and “glmnet” packages (Version 4.0–2) [26]. A nomogram was constructed by “Survival” and “rms” packages (Version 6.1–0) [27]. And a time-dependent ROC analysis was performed by the “survivalROC” package (Version 1.0.3) [28], C-index by “survival” package, and calibration curve by “rms” package. The ceRNA network was constructed by Cytoscape software. And we set a *p*-value lower than 0.05 as s statistical significance.

## Results

### Screening differentially expressed RNAs

**Fig. 1:**
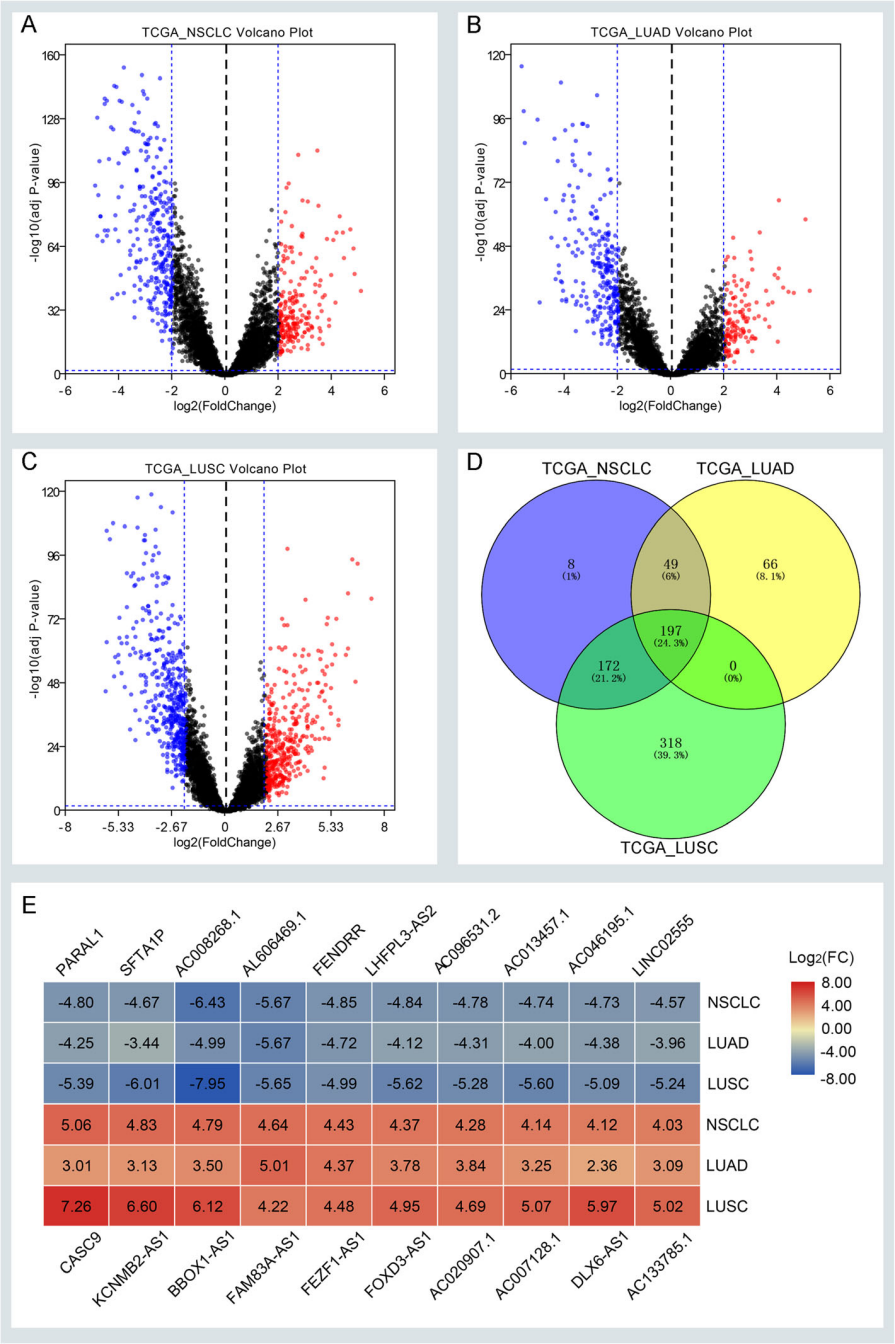
Screening differentially expressed lncRNAs (DELs) in three groups. **a–c** The volcano plots of DELs in the TCGA_NSCLC group, TCGA_LUAD group, and TCGA_LUSC group with thresholds of |log2FC| > 2, average expression > 2, and adjust *P*-value < 0.05, respectively. The red dots and blue dots represent the up-regulated and down-regulated DELs, separately. **d** The intersection of DELs in three groups. **e** The top 10 up and down DELs identified in three groups. FC, fold change

A total of 1145 NSCLC patients were enrolled from the TCGA database. We divided those data into three groups including the NSCLC group, LUAD group, and LUSC group. Then, we identified the DELs and DEMs in three groups by standards of |log2FC| > 2, average expression > 2, and adjust *P* value < 0.05, respectively. There are a total of 426 DELs in the NSCLC group, 312 DELs in the LUAD group, 687 DELs in the LUSC group, and 197 common DELs in three groups (Fig. 1a–d). There is a total of 1905 DEMs in the NSCLC group, 1434 DEMs in the LUAD group, 2641 DEMs in the LUSC group, and 1131 common DEMs in three groups (Supplementary Figure 1A–D). And there is a total of 130 DEMIs in the NSCLC group, 133 DEMIs in the LUAD group, 163 DEMIs in the LUSC group, and 89 common DEMIs in three groups (Supplementary Figure 5A–D) with the criteria of |log2FC| > 1, average expression > 1, and adjust *P*-value < 0.05. Also, we present the top 10 up and down-regulated DELs spotted in three groups (Fig. 1e). Common DELs in three groups were selected for the next survival analysis (Supplementary Table 5).

### Survival analysis

After excluding insufficient survival data or survival time less than 7 days, 970 NSCLC patients remained in our study. We displayed the clinical information of each group in Supplementary Table 1.

**Fig. 2:**
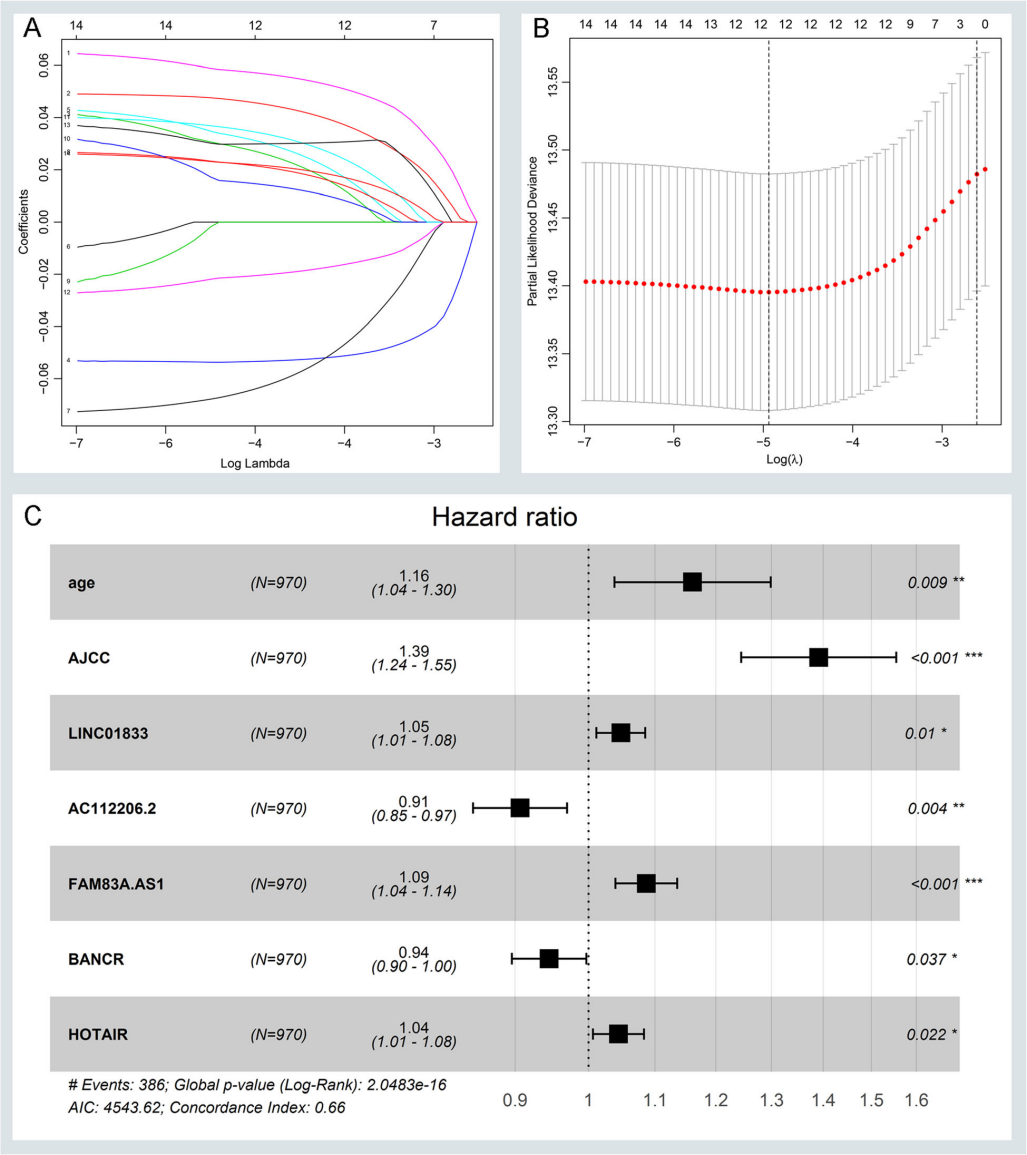
Identifying survival-related lncRNAs and building a risk score formula. Lasson analysis was applied to get the best cut-fit variables of the risk score formula. **a** LASSO coefficient profiles of all prognostic variables. **b** Validating the error rates of prognostic variables and calculating the best cut-fit variables. **c** Identifying and computing the most survival-related variables

**Fig. 3:**
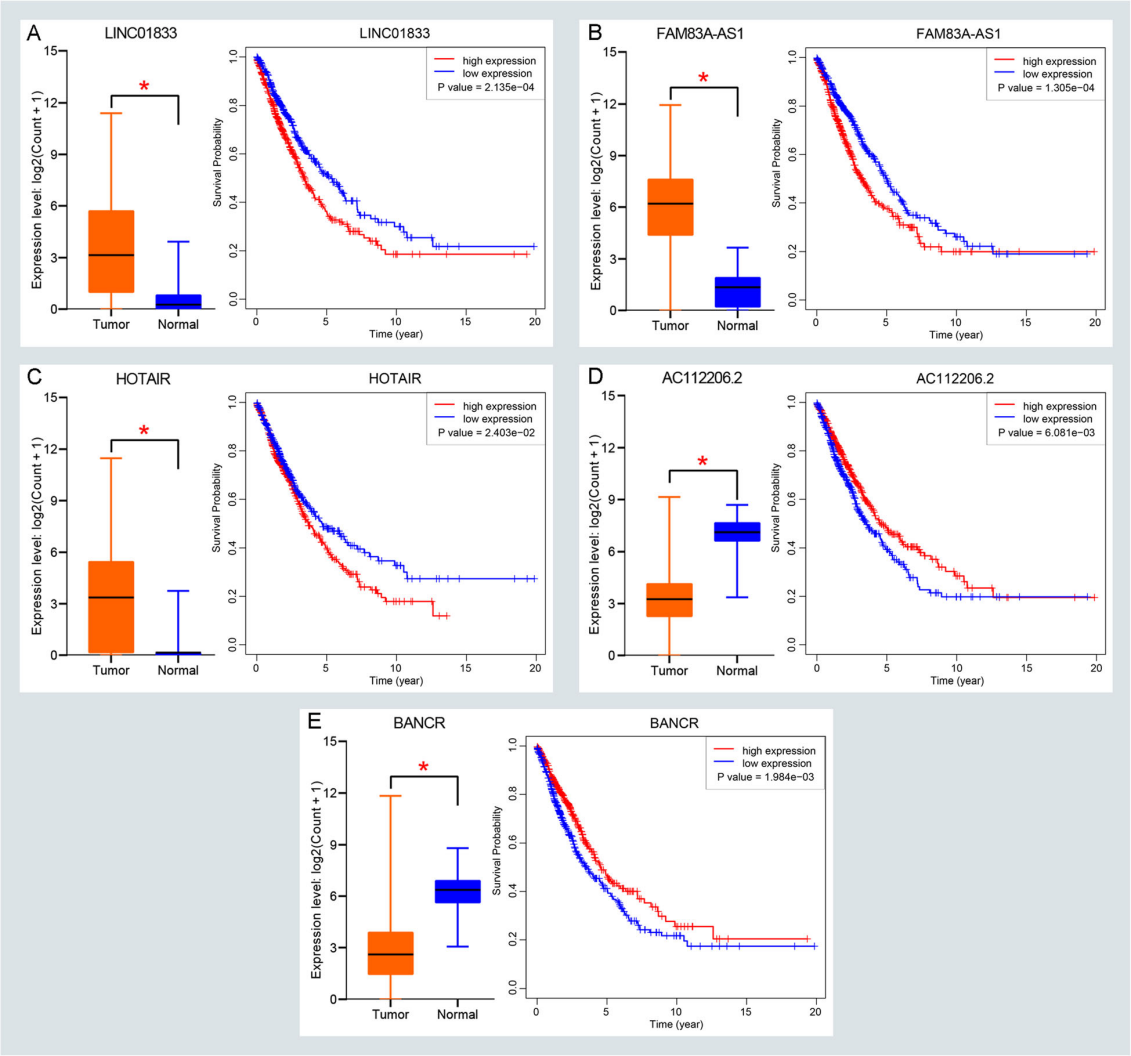
Screening and validating the expression roles and prognosis values of survival-related lncRNAs in NSCLC. **a**-**e** Validating expression roles and prognosis values of LINC01833, FAM83A-AS1, HOTAIR, AC112206.2, and BANCR in the NSCLC database, respectively. (*P < 0.05)

Briefly, the age of our cohort ranges from 33 to 88. There were 746 smoking patines among 970 NSCLC patients. And there were 23 EGFR mutations in the 98 patients and 24 KRAS mutations in the 68 patients. Additional details are available in Supplementary Table 1. We first performed univariate and multivariate CPHR to figure out the association between clinical parameters and OS. The results indicated that age and AJCC stage are independent survival factors as depicted in Supplementary Table 2. Next, univariable CPHR and Kaplan-Meier analysis were performed to assess the survival roles of common DELs. There a total of 14 lncRNAs that are not only related to OS but also show a statistical difference in univariable CPHR analysis with a *P*-value < 0.05. Combing with age and AJCC stage, a Lasson CPHR analysis was further applied to assess the best fitting variables in our cohort. The results suggested that only 12 variables that are best suitable for our further analysis as displayed in Fig. 2a-b. Furthermore, we assess the survival value of 12 variables through multivariable CPHR. Only 7 variables show statistic difference with a *P*-value < 0.05 as shown in Fig. 2c. We depicted the expression value and prognostic roles of 5 lncRNAs in Fig. 3a-e. There are 3 lncRNAs (LINC01833, FAM83A-AS1, and HOTAIR) that are not only high expression in the NSCLC group but also show worse survival. And there are only BANCR and AC112206.2 that are low expression with better survival. Ultimately, we identify five lncRNAs combined with age and AJCC stage which could predict the survival outcome of NSCLC patients.

### Developing a risk score formula and prognostic nomogram

**Fig. 4:**
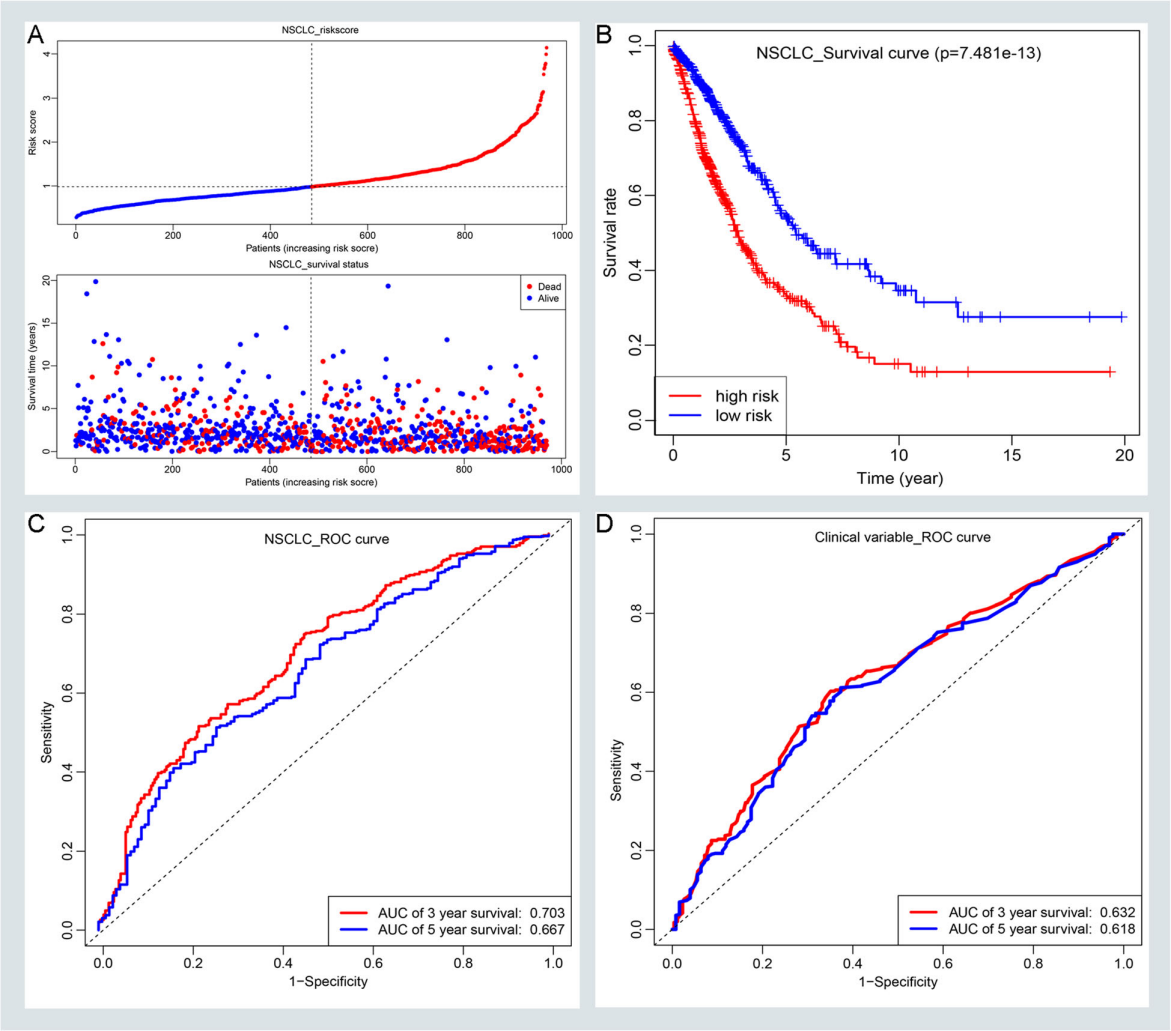
Assessing the prognostic performance of the risk score formula in the NSCLC group. **a** The risk score distribution and OS status of the formula in the NSCLC group. **b** Kaplan-Meier curves for OS based on the formula in the NSCLC group. The tick-marks on the curve represent the censored patients. **c** ROC curve analysis of the formula for predicting OS in the NSCLC group. **d** ROC curve analysis of the age and AJCC staging for predicting OS in the NSCLC group

**Fig. 5:**
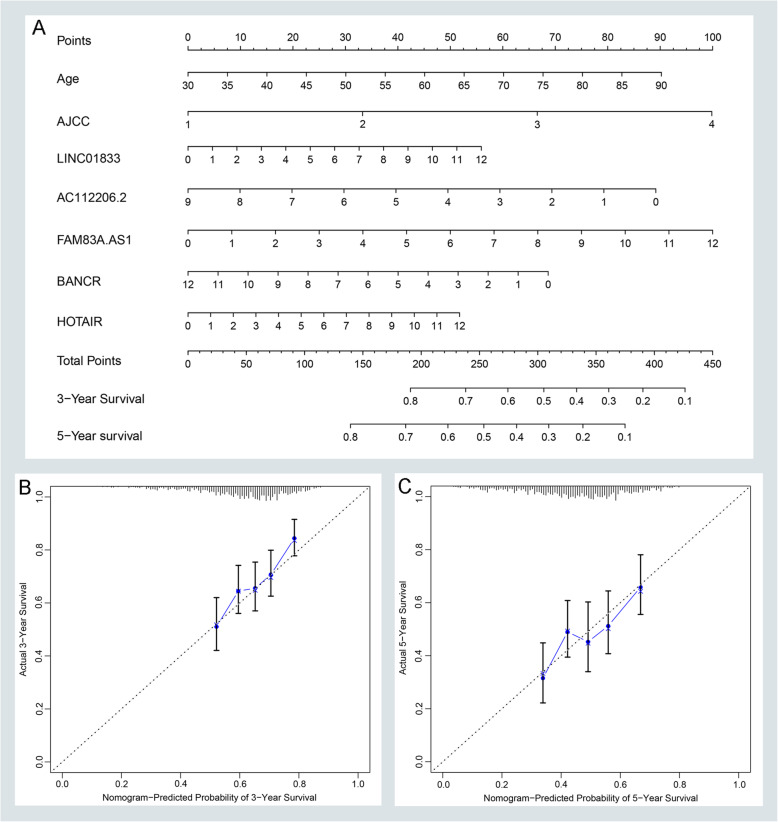
Nomogram construction and evaluation. **a** Nomogram to predict OS of patients with NSCLC. **b-c** Calibration curves of a nomogram to evaluate the prediction performance of 3-years and 5-years in the NSCLC group

Using multivariable CPHR analysis, we divided NSCLC patients into high-risk and low-risk groups based on the value of the median risk score. And we developed a risk score formula to understand the relationship between overall survival and lncRNAs with clinical variables. The formula was as shown as follows: Risk Score = (0.152 × age) + (0.370 × AJCC) + (0.127 × Expression LINC01833) + (0.250 × Expression FAM83A-AS1) + (0.127 × Expression HOTAIR) - (0.124 × Expression BANCR) - (0.283 × Expression AC112206.2). We depicted the distributions of risk score and the status of overall survival in the NSCLC group (Fig. 4a). Also, we validate the distributions of risk score and the status of overall survival in the LUAD group (Supplementary Figure 2A) and LUSC group (Supplementary Figure 2B). Furthermore, we found that the high-risk group of NSCLC patients had a worse prognostic outcome than patients with lower risk scores (Fig. 4b). And this tendency also appeared in the LUAD group (Supplementary Figure 2C) and LUSC group (Supplementary Figure 2D). We performed the time-related ROC curves to compare the sensitivities and specificities of predictive formula in NSCLC. The result suggested that the AUC value of 3 and 5 years was 0.703, 0.667, respectively (Fig. 4c). But the AUC value of clinical variables was only 0.632 and 0.618, which indicate that the prognostic prediction of our nomogram was better than the traditional age and AJCC stage (Fig. 4d). Also, we assessed the predictive ability of formula in LUAD and LUSC group. In the LUAD group, the AUC value of 3 and 5 years was 0.749, 0.735, respectively (Supplementary Figure 2E). The AUC value of 3 and 5 years was 0.65, 0.619 in the LUSC group, respectively (Supplementary Figure 2F). To vividly displayed the prognostic performance of OS-related variables, a nomogram was established. As shown in Fig. 5a, the nomogram could usefully predict the prognosis of 3 years and 5 years in NSCLC patients (Fig. 5a). Additionally, calibration curves suggested that the nomogram had a superior agreement between the predicted and actual OS of 3-year and 5-year in the NSCLC group (Fig. 5b-c) as well as in the LUAD group (Supplementary Figure 3A-B) and LUSC group (Supplementary Figure 3C-D). Moreover, we evaluated the relationship between our risk models and clinical variables. Our result indicated that the high-risk score of our risk model was related to age, male, smoking status, AJCC stage, and EGFR mutation with a statistical difference (*P* < 0.05). But there was no statistically significant difference in KRAS mutation (Supplementary Figure 6).

### Functional enrichment analysis

We performed the GO term and KEGG pathway analysis to detect the functional roles of 5 lncRNAs. We first calculated the correlation coefficient of 5 lncRNAs and selected DEMs with a correlation coefficient greater than 0.2 for enrichment analysis. There are 275 qualified DEMs in the correlation analysis of five lncRNAs. There are a total of 183 GO terms and 11 KEGG pathways that are primarily enriched in those DEMs including 119 terms in biological processes (BP), 37 terms in cellular components (CC), 27 terms in molecular functions (MF). We demonstrated the top ten BP, CC, MF, and KEGG Pathway in Supplementary Figure 4A–D. Briefly, the most enriched terms of GO were DNA metabolic process in BP, spindle in CC, and protein kinase binding in MF (Supplementary Figure 4A–C). Additionally, several cancer-related pathways were detected in the KEGG pathway analysis, for example, the p53 signaling pathway, MicroRNAs in cancer pathway, and Cell cycle pathway (Supplementary Figure 4D). Overall, the results from functional enrichment analysis were tightly linked with NSCLC.

### Construction of a ceRNA network

**Fig. 6:**
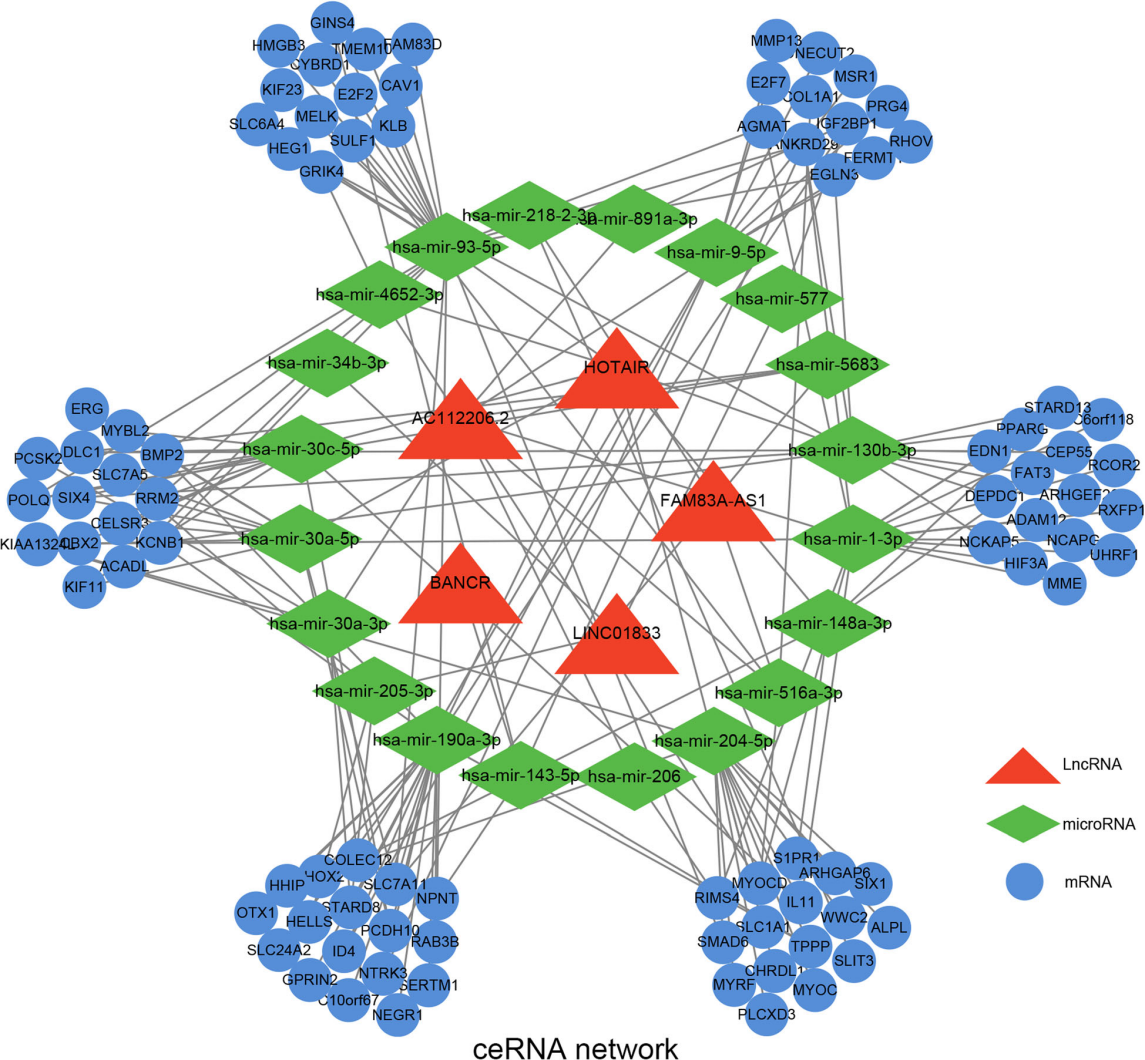
Schematic representations of 5 lncRNA-related ceRNA regulatory network in the progression of NSCLC

Based on the miRNA prediction from 5 lncRNAs, we identified 294 downstream miRNAs by the LncBase database (Supplementary Table 3). And we evaluated the expression of 294 miRNAs in the NSCLC group, LUAD group, and LUSC group. There are a total of 20 common miRNAs in four groups (Supplementary Figure 5D). Next, miRDB and miRTarBase databases were employed for screening miRNA-linked mRNAs. And we validated their expression in the DEMs of three groups. There are a total of 91 common mRNAs in four groups. According to prediction in Supplementary Table 4, we have found out 22 pairs of lncRNA-miRNA interactions, 145 pairs of miRNA-mRNA interactions. Finally, we constructed a ceRNA network in Fig. 6 to vividly display the interactions of 5 lncRNAs with 20 miRNAs and 91 mRNAs. Overall, this evidence has revealed that those lncRNAs not only effectively predict the survival outcome of NSCLC patients but also take part in the progression of NSCLC and potentially serve as a therapeutic target.

## Discussion

It is well acknowledged that the AJCC stage has been extensively used to estimate the survival outcome of tumor patients [29]. However, some limitations of the AJCC stage can be found in our clinical practice. For example, patients with similar anatomic sites and AJCC staging can exhibit variable responses to treatment and different survival outcomes. This difference may result from tumor heterogeneity, which partly arises from genetic mutations [30, 31]. Furthermore, recent studies have indicated that age and gender are also effective predictors for OS [32, 33]. So, we attempted to establish a new staging system that combines clinical variables with genetic mutations. Also, some evidence has indicated that lncRNAs not only act a regulatory role in the progression of NSCLC, but also have great potentials as biomarkers because they are stable, highly tissue-specific, and easy to detect in body fluids. For example, serum exosomal MALAT-1 was identified as a diagnostic predictor for NSCLC patients when they are in early-phase or metastasis [13]. High expression of HOTAIR was closely related to progressive disease, worse survival, and more potential in tumor recurrence after radical operation [34]. Low expression of GAS6-AS1 was connected with the occurrence of lymph node metastasis and an independent biomarker for the prognostic outcome of NSCLC [35]. Therefore, it is urgent to set up a reliable prognostic model for NSCLC.

In this research, we successfully identified a survival nomogram based on lncRNAs and clinical variables of NSCLC. We combined age and AJCC staging with 5 lncRNAs into a risk formula and weighted each parameter to detect their relationship with overall survival. And a nomogram was established to vividly quantify the OS probability of each variable. Notably, we weighted each lncRNA into a nomogram instead of integrating 5 lncRNAs as a whole [36, 37]. Because it is difficult to test all variables at a time in clinical practice. In our nomogram, we weighted a risk point to every variable and calculated patient survival at 3 or 5 years based on the total risk point. And the predictive performance of our nomogram was superior to the traditional age and AJCC stage. Moreover, our nomogram is easy to understand. Its simplicity will allow clinicians to quickly evaluate survival outcomes and make decisions about individual NSCLC patients. Even individuals without a medical background can easily understand the meaning of our nomogram. Those features will make our nomogram an accurate and effective biomarker for clinical applications.

Additionally, the functional analysis indicated that 5 lncRNAs were also involved in several cancer pathways, for example, p53 and MicroRNAs in cancer pathways. Moreover, some studies have found their role in the progression of NSCLC. The high expression of HOTAIR is involved in many types of biological processes in NSCLC. For instance, HOTAIR contributes to the down-regulating expression of p21WAF1/CIP1, thereby inducing the cisplatin resistance of A549 cells [38]. Wang et al. demonstrated that HOTAIR was reported to facilitate the proliferation and migration of A549 and H838 cells through sponging with miR-326, thus control the expression of phox2a [39]. The high expression of HOTAIR can be controlled by Col-1, thereby promoting the formation of microenvironment and progression of NSCLC [40]. Several studies have recently revealed that the lower expression of BANCR was related to the initiation and progression of NSCLC. Sun et al. found that lower expression of BANCR can encourage the epithelial-mesenchymal transition of A549 and SPC-A1 cells and improve their ability in invasion and metastasis [41]. Up-regulating BANCR was tightly linked with radiotherapy for lung cancer [42]. Jiang et al. discovered that BANCR was able to moderate the ability of invasion and metastasis in lung cancer through the p38 MAPK and JNK pathway rather than the ERK MAPK pathway [43]. LINC01833 was demonstrated to promote the infiltration and metastasis of LUAD by adsorbing miR-519e-3p through a sponge and regulate S100A4 expression [44]. FAM83A-AS1 was proved to enhance cell migration, invasion and EMT by modulating the miR-150-5p/MMP14 pathway [45]. So, the lncRNAs in the nomogram can not only serve as a prognostic biomarker but also function as a regulator in the occurrence and progression of tumors. Notably, those lncRNAs can act as a ceRNA network, thereby participating in cancer progression. For instance, HOTAIR/miR-149-5p/HNRNPA1 axis promotes the cell growth, migration, and invasion in NSCLC [46]; FAM83A-AS1/miR-150-5p/MMP14 regulates LUAD progression and invasion [45]; BANCR/miR-338-3p/IGF1R network regulated Raf/MEK/ERK pathway, thereby encouraging the proliferation, migration, invasion and epithelial-mesenchymal transition (EMT) of esophageal cancer [47]. Those studies indicated that the lncRNAs in the nomogram can function as a therapeutic target for NSCLC.

Although our lncRNAs-related nomogram showed good performance in survival prediction of NSCLC, some limitations can be detected in our research. First, our study is a retrospective study. But 970 patients is a large sample. So our results are reliable. Additionally, our data lacked information such as chemo-radiotherapy history, smoking history, and patients’ disease history. This may result from the limitations of our data. So, further clinical studies are needed to verify our results when applied to clinical practice. Last, we identified survival-related lncRNAs to construct the nomogram, which might overlook some valuable information. All in all, despite these limitations in our study, we believe that our persistent efforts will eventually establish an ideal prognostic model in clinical practice.

## Conclusions

In summary, by a large sample analysis, we successfully constructed a nomogram based on lncRNAs and clinical variables that predicts the survival of NSCLC patients. And the predictive performance of our prognostic nomogram was better than the traditional AJCC stage and age. In addition to the survival prediction of our nomogram, functional analysis and ceRNA network also indicate that they might involve in cancer progression and potentially serve as a therapeutic target for NSCLC.

## Supplementary Information

**Additional file 1 **: **Supplementary Figure 1.** Screening differentially expressed mRNAs (DEMs) in three groups. (A–C) The volcano plots of DEMs in the TCGA_NSCLC group, TCGA_LUAD group, and TCGA_LUSC group with thresholds of |log2FC| > 2, average expression > 2, and adjust *P*-value < 0.05, respectively. The red dots and blue dots represent the up-regulated and down-regulated DEMs, separately. (D) The intersection of DELs in three groups.

**Additional file 2 **: **Supplementary Figure 2.** Assessing the prognostic performance of the risk score formula in LUAD and LUSC group. (A-B) The risk score distribution and OS status of the formula in LUAD and LUSC group, respectively. (C-D) Kaplan-Meier curves for OS based on the formula in LUAD and LUSC group, separately. The tick-marks on the curve represent the censored patients. (E-F) ROC curve analysis of the formula for predicting OS in LUAD and LUSC group, respectively.

**Additional file 3 **: **Supplementary Figure 3.** Evaluating the prediction performance of the nomogram. (A-B) Calibration curves of a nomogram to evaluate the prediction performance of 3-years and 5-years in the LUAD group. (C-D) Calibration curves of a nomogram to evaluate the prediction performance of 3-years and 5-years in the LUSC group.

**Additional file 4 **: **Supplementary Figure 4.** Functional enrichment analysis for the differentially expressed mRNAs (DEMs) of 5 lncRNAs. (A-C) The top ten enriched GO terms of qualified DEMs with a correlation coefficient greater than 0.2 in biological processes (BP), cellular components (CC), and molecular functions (MF), respectively. (D) The top ten enriched KEGG pathways of qualified DEMs in the NSCLC database.

**Additional file 5 **: **Supplementary Figure 5.** Screening differentially expressed miRNA (DEMIs) in three groups. (A–C) The volcano plots of DEMIs in the TCGA_NSCLC group, TCGA_LUAD group, and TCGA_LUSC group with thresholds of |log2FC| > 1, average expression > 1, and adjust *P*-value < 0.05, respectively. The red dots and blue dots represent the up-regulated and down-regulated DEMIs, separately. (D) The intersection of DEMIs in three groups and the LncBase database.

**Additional file 6 **: **Supplementary Figure 6.** The relationship between risk models and clinical variables. (A-E) High-risk score of our risk model was related to age (*P* < 0.001), male (*P* < 0.001), smoking status (*P* = 0.0165), AJCC stage (*P* < 0.001), and EGFR mutation (*P* = 0.0063) with a statistical difference. (F) But there was no statistically significant difference in KRAS mutation (*P* = 0.3895).

**Additional file 7 **: **Supplementary Table 1**. Clinical characteristics of NSCLC patients in this study.

**Additional file 8 **: **Supplementary Table 2.** Univariate and multivariate Cox regression analysis in NSCLC.

**Additional file 9 **: **Supplementary Table 3.** The lncRNA-miRNA pairs were predicted by the LncBase database.

**Additional file 10 **: **Supplementary Table 4.** The qualified lncRNA-miRNA and miRNA-mRNA pairs were constructed in the ceRNA network.

**Additional file 11 **: **Supplementary Table 5.** Differentially expressed RANs in our analysis.

## Data Availability

The datasets generated during the current study are available in The Cancer Genome Atlas (TCGA) program (https://portal.gdc.cancer.gov/repository).

## Abbreviations

NSCLC: Non-small cell lung cancer
LUAD: Lung adenocarcinoma
LUSC: Lung squamous carcinoma
lncRNAs: Long non-coding RNAs
CPHR: Cox proportional hazards regression
TCGA: The Cancer Genome Atlas
GO: Gene Ontology
KEGG: Kyoto Encyclopedia of Gene and Genomes
AJCC: American Joint Committee on Cancer
OS: Overall survival
HR: Hazard ratio
CI: Confidence interval
ROC: Receiver operating curves
AUC: Area Under Curve
ceRNA: Competing endogenous RNA
