# Supplementary figures and images for: Five long non-coding RNAs establish a prognostic nomogram and construct a competing endogenous RNA network in the progression of non-small cell lung cancer
Source: BMC Cancer. 2021 Apr 23;21:457. doi: 10.1186/s12885-021-08207-7 (PMC8067646; doi:10.1186/s12885-021-08207-7)

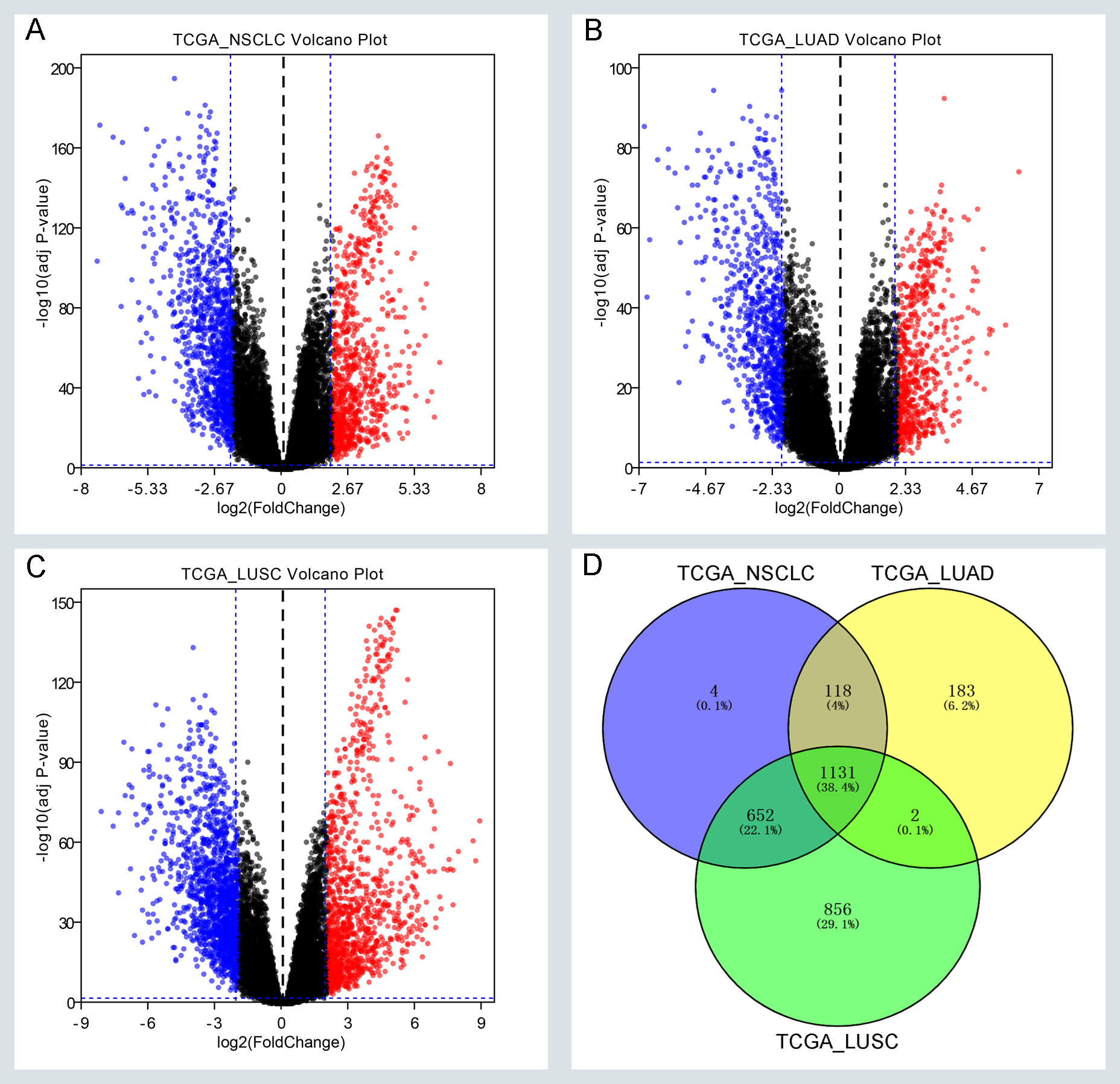

Supplement: Supplementary file 1 — Additional file 1 : Supplementary Figure 1. Screening differentially expressed mRNAs (DEMs) in three groups. (A–C) The volcano plots of DEMs in the TCGA_NSCLC group, TCGA_LUAD group, and TCGA_LUSC group with thresholds of |log2FC| > 2, average expression > 2, and adjust P-value < 0.05, respectively. The red dots and blue dots represent the up-regulated and down-regulated DEMs, separately. (D) The intersection of DELs in three groups. [file 12885_2021_8207_MOESM1_ESM.tif]

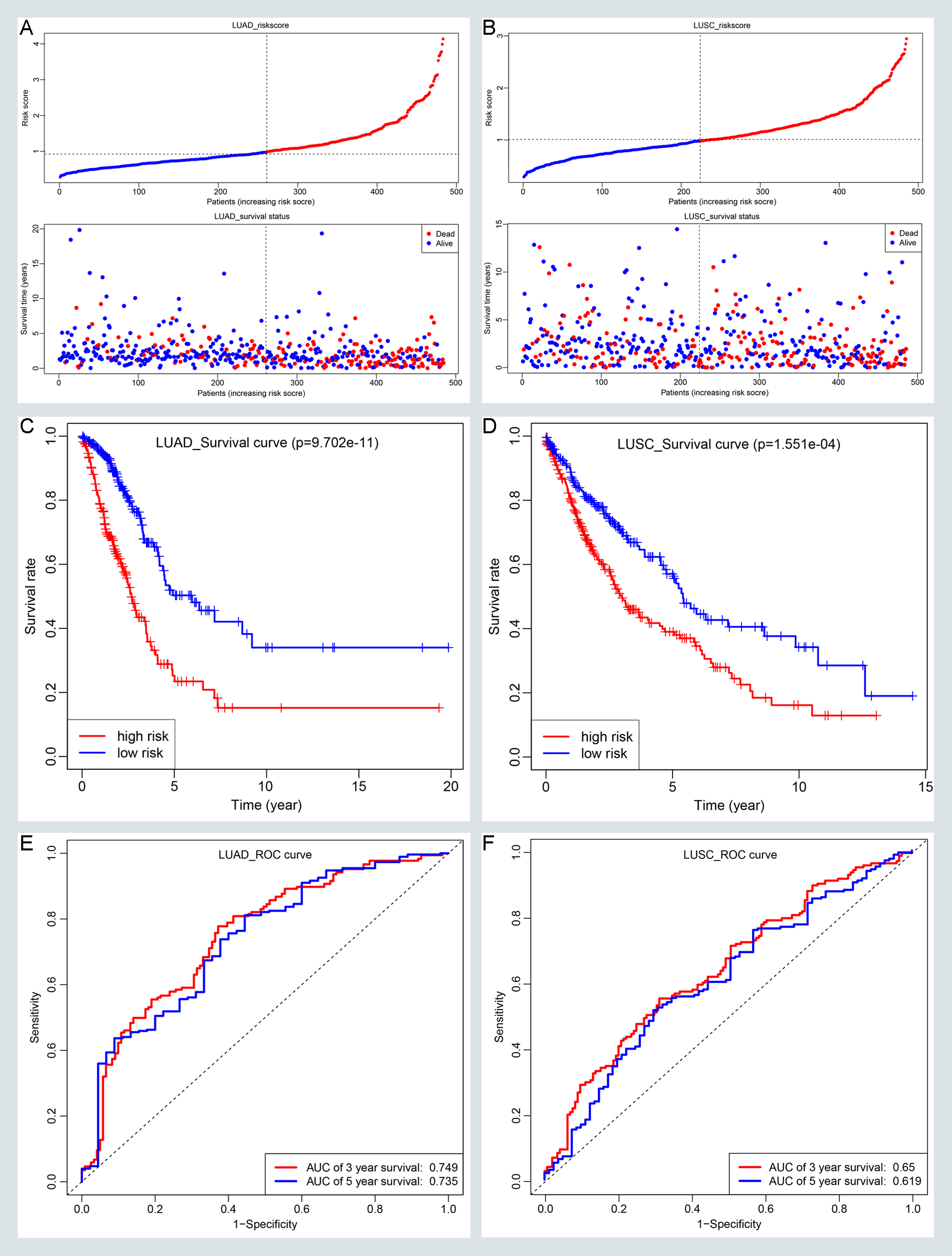

Supplement: Supplementary file 2 — Additional file 2 : Supplementary Figure 2. Assessing the prognostic performance of the risk score formula in LUAD and LUSC group. (A-B) The risk score distribution and OS status of the formula in LUAD and LUSC group, respectively. (C-D) Kaplan-Meier curves for OS based on the formula in LUAD and LUSC group, separately. The tick-marks on the curve represent the censored patients. (E-F) ROC curve analysis of the formula for predicting OS in LUAD and LUSC group, respectively. [file 12885_2021_8207_MOESM2_ESM.tif]

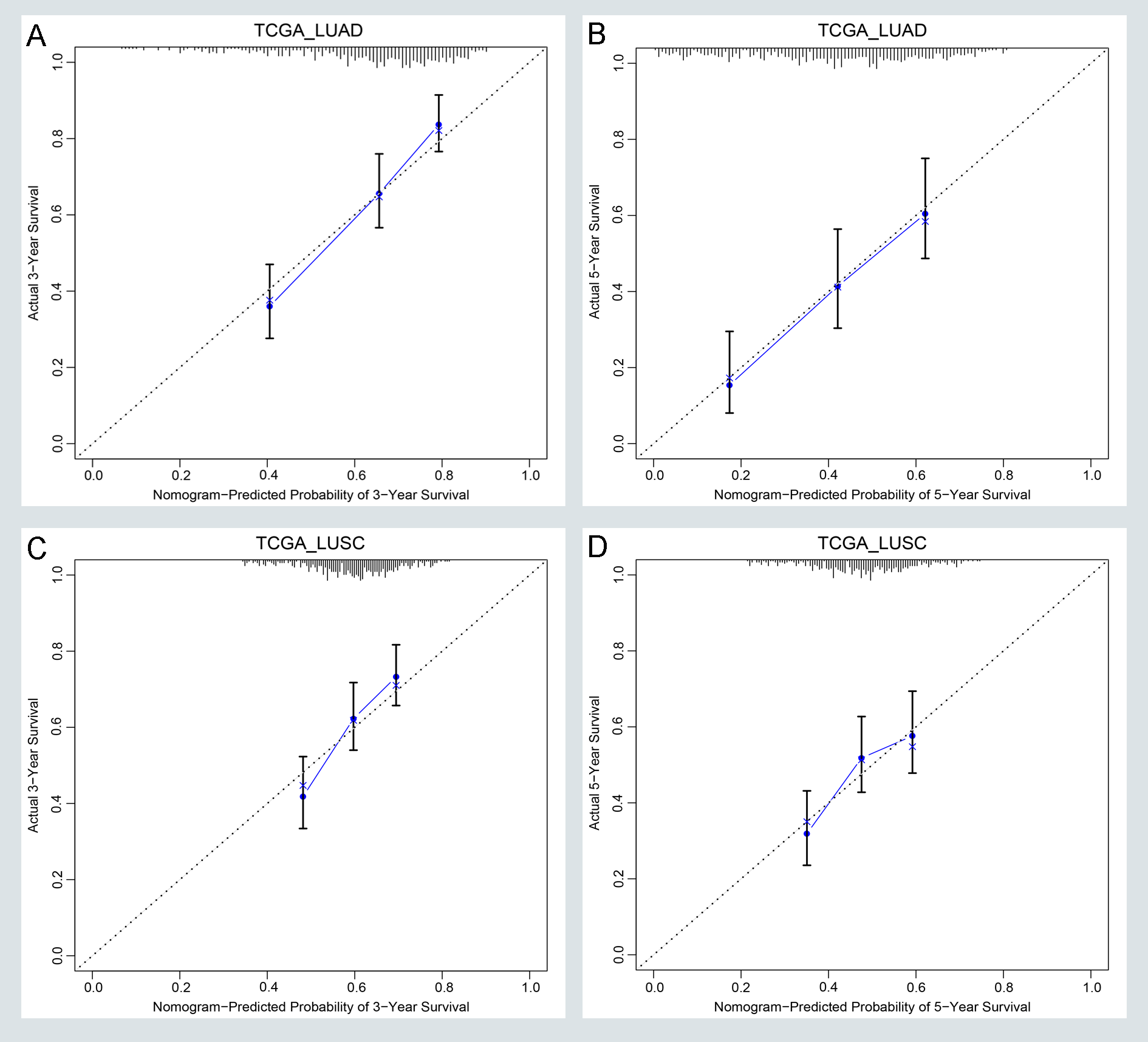

Supplement: Supplementary file 3 — Additional file 3 : Supplementary Figure 3. Evaluating the prediction performance of the nomogram. (A-B) Calibration curves of a nomogram to evaluate the prediction performance of 3-years and 5-years in the LUAD group. (C-D) Calibration curves of a nomogram to evaluate the prediction performance of 3-years and 5-years in the LUSC group. [file 12885_2021_8207_MOESM3_ESM.tif]

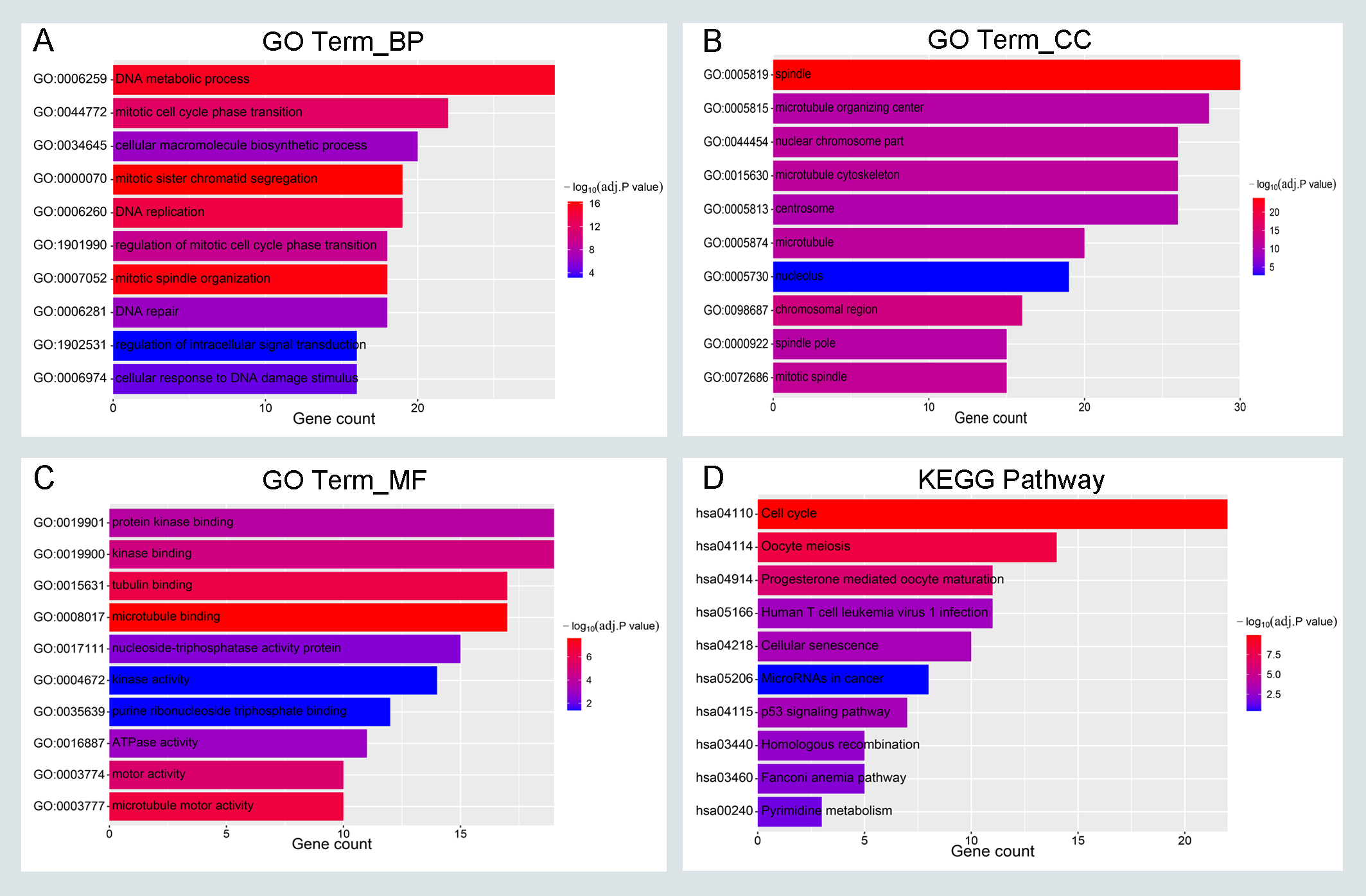

Supplement: Supplementary file 4 — Additional file 4 : Supplementary Figure 4. Functional enrichment analysis for the differentially expressed mRNAs (DEMs) of 5 lncRNAs. (A-C) The top ten enriched GO terms of qualified DEMs with a correlation coefficient greater than 0.2 in biological processes (BP), cellular components (CC), and molecular functions (MF), respectively. (D) The top ten enriched KEGG pathways of qualified DEMs in the NSCLC database. [file 12885_2021_8207_MOESM4_ESM.tif]

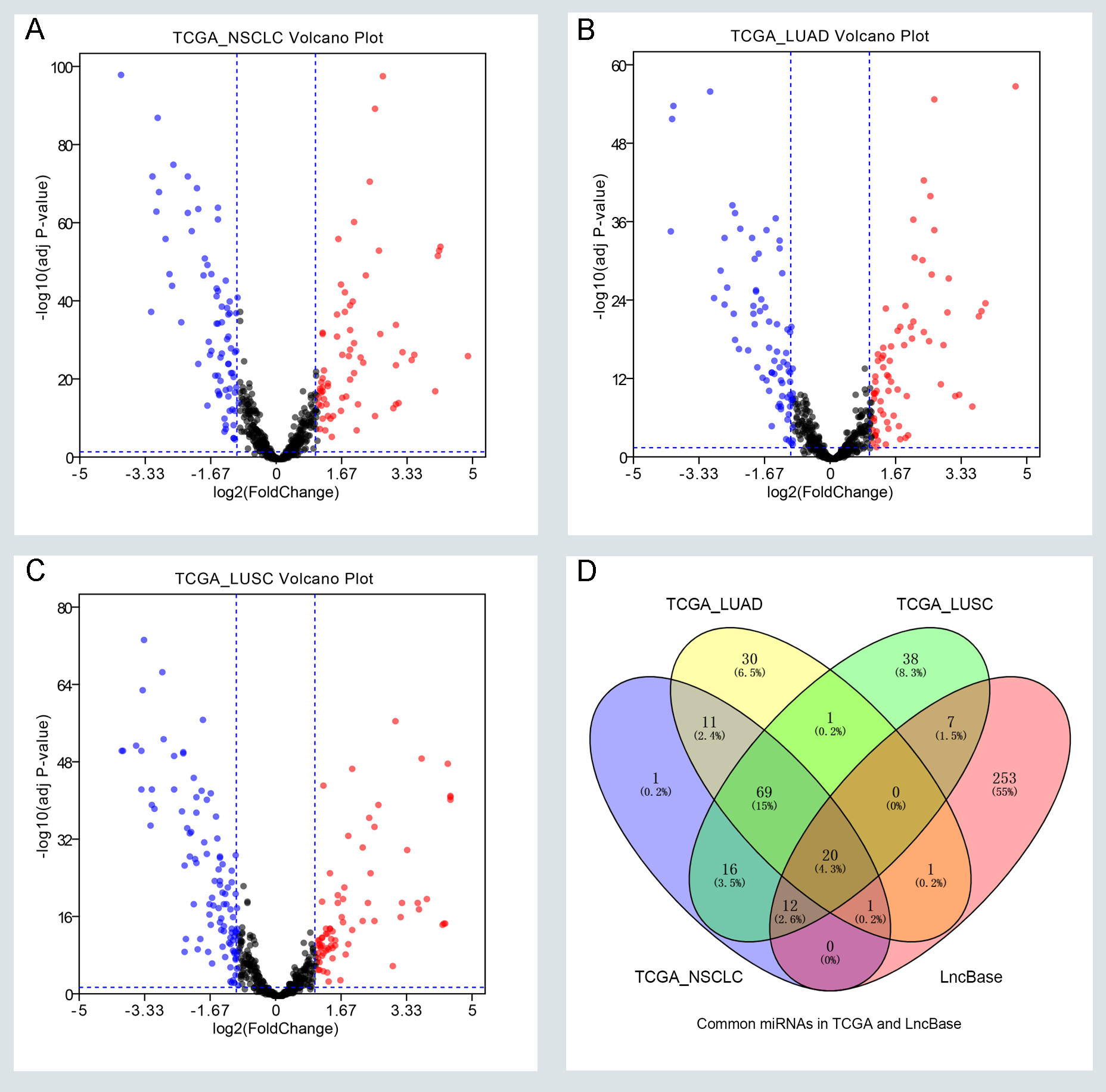

Supplement: Supplementary file 5 — Additional file 5 : Supplementary Figure 5. Screening differentially expressed miRNA (DEMIs) in three groups. (A–C) The volcano plots of DEMIs in the TCGA_NSCLC group, TCGA_LUAD group, and TCGA_LUSC group with thresholds of |log2FC| > 1, average expression > 1, and adjust P-value < 0.05, respectively. The red dots and blue dots represent the up-regulated and down-regulated DEMIs, separately. (D) The intersection of DEMIs in three groups and the LncBase database. [file 12885_2021_8207_MOESM5_ESM.tif]

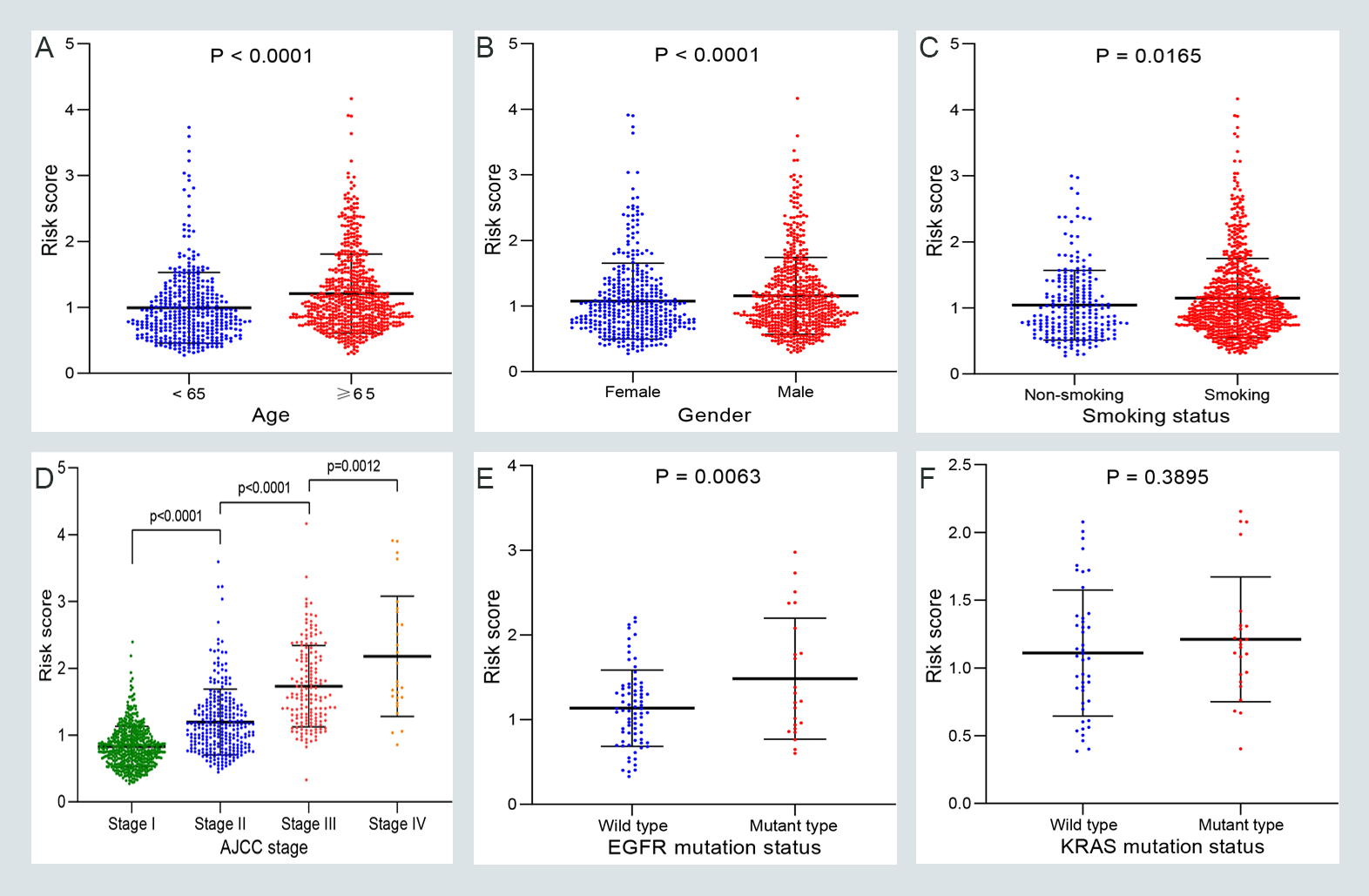

Supplement: Supplementary file 6 — Additional file 6 : Supplementary Figure 6. The relationship between risk models and clinical variables. (A-E) High-risk score of our risk model was related to age (P < 0.001), male (P < 0.001), smoking status (P = 0.0165), AJCC stage (P < 0.001), and EGFR mutation (P = 0.0063) with a statistical difference. (F) But there was no statistically significant difference in KRAS mutation (P = 0.3895). [file 12885_2021_8207_MOESM6_ESM.tif]
