# Supplementary material for: Five long non-coding RNAs establish a prognostic nomogram and construct a competing endogenous RNA network in the progression of non-small cell lung cancer
Source: BMC Cancer. 2021 Apr 23;21:457. doi: 10.1186/s12885-021-08207-7 (PMC8067646; doi:10.1186/s12885-021-08207-7)
Supplement: Supplementary file 7 — Additional file 7 : Supplementary Table 1. Clinical characteristics of NSCLC patients in this study. [file 12885_2021_8207_MOESM7_ESM.docx]

Table S1. Clinical characteristics of NSCLC patients in this study.

| Characteristic | NSCLC dataset | LUAD dataset | LUSC dataset | P Value |
| --- | --- | --- | --- | --- |
|  | n=970 | n=484 | n=486 |  |
| **Age (years)** |  |  |  | 0.002 |
| ≥65 | 587 | 269 (45.83%) | 318 (54.17%) |  |
| <65 | 383 | 215 (56.14%) | 168 (43.86%) |  |
| **Gender** |  |  |  | 0.001 |
| Female | 390 | 263 (67.44%) | 127 (32.56%) |  |
| Male | 580 | 221 (38.1%) | 359 (61.9%) |  |
| **Smoking status** |  |  |  | 0.001 |
| Smoking | 746 | 335 (69.2%) | 411 (84.6%) |  |
| Not smoking | 224 | 149 (30.8%) | 75 (15.4%) |  |
| **EGFR mutation** |  |  |  | 0.089 |
| Positive | 23 | 21 (27.3%) | 2 (9.5%) |  |
| Negative | 75 | 56 (72.7%) | 19 (90.5%) |  |
| NA | 872 |  |  |  |
| **KRAS mutation** |  |  |  | 0.070 |
| Positive | 24 | 23 (39.7%) | 1 (10.0%) |  |
| Negative | 44 | 35 (60.3%) | 9 (90.0%) |  |
| NA | 902 |  |  |  |
| **AJCC stage** |  |  |  | 0.448 |
| I + II | 779 | 384 (49.29%) | 395 (50.71%) |  |
| III + IV | 191 | 100 (52.36%) | 91 (47.64%) |  |
| **Tumor stage** |  |  |  | 0.009 |
| T1 + T2 | 816 | 422 (51.72%) | 394 (48.28%) |  |
| T3 + T4 | 154 | 62 (40.26%) | 92 (59.74%) |  |
| **Lymph metastasis** |  |  |  | 0.574 |
| N0  N1 + N2 | 637  333 | 322 (50.55%)  171 (51.35%) | 315 (49.45%)  164 (49.25%) |  |
| **Distant metastasis** |  |  |  | 0.003 |
| M0 | 943 | 463 (49.1%) | 480 (50.9%) |  |
| M1 | 27 | 21 (77.78%) | 6 (22.22%) |  |

Abbreviations: NSCLC, non-small cell lung cancer; LUAD, lung adenocarcinoma; LUSC, lung squamous carcinoma; EGFR, epidermal growth factor receptor; KRAS, Kirsten rat sarcoma 2 viral oncogene homolog; AJCC, the American Joint Committee on Cancer.
