# Supplementary material for: Five long non-coding RNAs establish a prognostic nomogram and construct a competing endogenous RNA network in the progression of non-small cell lung cancer
Source: BMC Cancer. 2021 Apr 23;21:457. doi: 10.1186/s12885-021-08207-7 (PMC8067646; doi:10.1186/s12885-021-08207-7)
Supplement: Supplementary file 8 — Additional file 8 : Supplementary Table 2. Univariate and multivariate Cox regression analysis in NSCLC. [file 12885_2021_8207_MOESM8_ESM.docx]

Table S2. Univariate and multivariate Cox regression analysis in NSCLC.

| Characteristic | Univariate analysis | |  | Multivariate analysis | |
| --- | --- | --- | --- | --- | --- |
|  | HR (95% CI) | P value |  | HR (95% CI) | P value |
| Age | 1.012 (1.001-1.024) | 0.034 |  | 1.015 (1.004-1.027) | 0.008 |
| Gender | 0.841 (0.683-1.036) | 0.103 |  |  |  |
| Smoke | 0.946(0.744−1.203) | 0.651 |  |  |  |
| AJCC stage | 1.431 (1.283-1.595) | < 0.001 |  | 1.451 (1.301-1.619) | < 0.001 |
| Tumor stage | 1.396 (1.23-1.586) | < 0.001 |  |  |  |
| Lymph node metastasis | 1.401 (1.227-1.6) | < 0.001 |  |  |  |
| Distant metastasis | 1.98 (1.216-3.225) | 0.006 |  |  |  |

Abbreviations: NSCLC, non-small cell lung cancer; AJCC, the American Joint Committee on Cancer.
