# Supplementary material for: Five long non-coding RNAs establish a prognostic nomogram and construct a competing endogenous RNA network in the progression of non-small cell lung cancer
Source: BMC Cancer. 2021 Apr 23;21:457. doi: 10.1186/s12885-021-08207-7 (PMC8067646; doi:10.1186/s12885-021-08207-7)
Supplement: Supplementary file 9 — Additional file 9 : Supplementary Table 3. The lncRNA-miRNA pairs were predicted by the LncBase database. [file 12885_2021_8207_MOESM9_ESM.docx]

**Table S3**. The lncRNA-miRNA pairs predicted by the LncBase database.

| lncRNA | miRNA | Prediction score |
| --- | --- | --- |
| FAM83A-AS1 | hsa-mir-150 | 0.976 |
| FAM83A-AS1 | hsa-mir-1226 | 0.976 |
| FAM83A-AS1 | hsa-mir-4325 | 0.975 |
| FAM83A-AS1 | hsa-mir-942 | 0.972 |
| FAM83A-AS1 | hsa-mir-588 | 0.955 |
| FAM83A-AS1 | hsa-mir-4713 | 0.95 |
| FAM83A-AS1 | hsa-mir-3918 | 0.937 |
| FAM83A-AS1 | hsa-mir-7162 | 0.931 |
| FAM83A-AS1 | hsa-mir-597 | 0.93 |
| FAM83A-AS1 | hsa-mir-629 | 0.927 |
| FAM83A-AS1 | hsa-mir-516a | 0.923 |
| FAM83A-AS1 | hsa-mir-516b | 0.923 |
| FAM83A-AS1 | hsa-mir-4781 | 0.893 |
| FAM83A-AS1 | hsa-mir-541 | 0.892 |
| FAM83A-AS1 | hsa-mir-6835 | 0.889 |
| FAM83A-AS1 | hsa-mir-6866 | 0.886 |
| FAM83A-AS1 | hsa-mir-4766 | 0.883 |
| FAM83A-AS1 | hsa-mir-4659b | 0.88 |
| FAM83A-AS1 | hsa-mir-4659a | 0.88 |
| FAM83A-AS1 | hsa-mir-5196 | 0.88 |
| FAM83A-AS1 | hsa-mir-4459 | 0.877 |
| FAM83A-AS1 | hsa-mir-4435 | 0.869 |
| FAM83A-AS1 | hsa-mir-221 | 0.866 |
| FAM83A-AS1 | hsa-mir-6893 | 0.865 |
| FAM83A-AS1 | hsa-mir-1178 | 0.862 |
| FAM83A-AS1 | hsa-mir-6836 | 0.862 |
| FAM83A-AS1 | hsa-mir-4512 | 0.862 |
| FAM83A-AS1 | hsa-mir-218 | 0.861 |
| FAM83A-AS1 | hsa-mir-3074 | 0.856 |
| FAM83A-AS1 | hsa-mir-5001 | 0.855 |
| FAM83A-AS1 | hsa-mir-5691 | 0.853 |
| FAM83A-AS1 | hsa-mir-938 | 0.848 |
| FAM83A-AS1 | hsa-mir-4446 | 0.846 |
| FAM83A-AS1 | hsa-mir-5582 | 0.842 |
| FAM83A-AS1 | hsa-mir-3619 | 0.842 |
| FAM83A-AS1 | hsa-mir-370 | 0.841 |
| FAM83A-AS1 | hsa-mir-6837 | 0.826 |
| FAM83A-AS1 | hsa-mir-3944 | 0.826 |
| FAM83A-AS1 | hsa-mir-6783 | 0.815 |
| FAM83A-AS1 | hsa-mir-6733 | 0.815 |
| FAM83A-AS1 | hsa-mir-1200 | 0.814 |
|  |  |  |
| FAM83A-AS1 | hsa-mir-378g | 0.814 |
| FAM83A-AS1 | hsa-mir-3185 | 0.814 |
| FAM83A-AS1 | hsa-mir-545 | 0.812 |
| FAM83A-AS1 | hsa-mir-653 | 0.805 |
| FAM83A-AS1 | hsa-mir-4505 | 0.803 |
| FAM83A-AS1 | hsa-mir-6895 | 0.802 |
| FAM83A-AS1 | hsa-mir-5787 | 0.8 |
| HOTAIR | hsa-mir-6802 | 1 |
| HOTAIR | hsa-mir-4743 | 0.998 |
| HOTAIR | hsa-mir-3622b | 0.994 |
| HOTAIR | hsa-mir-3622a | 0.993 |
| HOTAIR | hsa-mir-6511b | 0.986 |
| HOTAIR | hsa-mir-3688 | 0.971 |
| HOTAIR | hsa-mir-3148 | 0.969 |
| HOTAIR | hsa-mir-5011 | 0.966 |
| HOTAIR | hsa-mir-302f | 0.965 |
| HOTAIR | hsa-mir-548ar | 0.964 |
| HOTAIR | hsa-mir-129 | 0.964 |
| HOTAIR | hsa-mir-3190 | 0.961 |
| HOTAIR | hsa-mir-4677 | 0.96 |
| HOTAIR | hsa-mir-6817 | 0.959 |
| HOTAIR | hsa-mir-6879 | 0.957 |
| HOTAIR | hsa-mir-3127 | 0.956 |
| HOTAIR | hsa-mir-4801 | 0.929 |
| HOTAIR | hsa-mir-197 | 0.924 |
| HOTAIR | hsa-mir-6735 | 0.922 |
| HOTAIR | hsa-mir-326 | 0.92 |
| HOTAIR | hsa-mir-6079 | 0.918 |
| HOTAIR | hsa-mir-5006 | 0.913 |
| HOTAIR | hsa-mir-6833 | 0.91 |
| HOTAIR | hsa-mir-29b | 0.91 |
| HOTAIR | hsa-mir-30c | 0.907 |
| HOTAIR | hsa-mir-6165 | 0.907 |
| HOTAIR | hsa-mir-30a | 0.904 |
| HOTAIR | hsa-mir-30e | 0.904 |
| HOTAIR | hsa-mir-518c | 0.902 |
| HOTAIR | hsa-mir-136 | 0.901 |
| HOTAIR | hsa-mir-3663 | 0.901 |
| HOTAIR | hsa-mir-6873 | 0.9 |
| HOTAIR | hsa-mir-4722 | 0.899 |
| HOTAIR | hsa-mir-30d | 0.899 |
| HOTAIR | hsa-mir-30b | 0.897 |
| HOTAIR | hsa-mir-4667 | 0.894 |
| HOTAIR | hsa-mir-4731 | 0.88 |
| HOTAIR | hsa-mir-3182 | 0.88 |
| HOTAIR | hsa-mir-1277 | 0.876 |
| HOTAIR | hsa-mir-6808 | 0.872 |
| HOTAIR | hsa-mir-511 | 0.872 |
| HOTAIR | hsa-mir-4436b | 0.872 |
| HOTAIR | hsa-mir-330 | 0.866 |
| HOTAIR | hsa-mir-190a | 0.864 |
| HOTAIR | hsa-mir-3910 | 0.864 |
| HOTAIR | hsa-mir-4326 | 0.863 |
| HOTAIR | hsa-mir-4318 | 0.862 |
| HOTAIR | hsa-mir-627 | 0.856 |
| HOTAIR | hsa-mir-4666a | 0.855 |
| HOTAIR | hsa-mir-3666 | 0.853 |
| HOTAIR | hsa-mir-211 | 0.851 |
| HOTAIR | hsa-mir-4755 | 0.85 |
| HOTAIR | hsa-mir-17 | 0.848 |
| HOTAIR | hsa-mir-6772 | 0.848 |
| HOTAIR | hsa-mir-148a | 0.846 |
| HOTAIR | hsa-mir-4498 | 0.845 |
| HOTAIR | hsa-mir-7110 | 0.844 |
| HOTAIR | hsa-mir-548av | 0.841 |
| HOTAIR | hsa-mir-4692 | 0.838 |
| HOTAIR | hsa-mir-519d | 0.834 |
| HOTAIR | hsa-mir-1273g | 0.833 |
| HOTAIR | hsa-mir-586 | 0.832 |
| HOTAIR | hsa-mir-93 | 0.831 |
| HOTAIR | hsa-mir-6764 | 0.83 |
| HOTAIR | hsa-mir-126 | 0.829 |
| HOTAIR | hsa-mir-20b | 0.829 |
| HOTAIR | hsa-mir-204 | 0.828 |
| HOTAIR | hsa-mir-5189 | 0.827 |
| HOTAIR | hsa-mir-6786 | 0.825 |
| HOTAIR | hsa-mir-7843 | 0.825 |
| HOTAIR | hsa-mir-3157 | 0.825 |
| HOTAIR | hsa-mir-6867 | 0.824 |
| HOTAIR | hsa-mir-6824 | 0.823 |
| HOTAIR | hsa-mir-134 | 0.822 |
| HOTAIR | hsa-mir-3684 | 0.819 |
| HOTAIR | hsa-mir-4768 | 0.819 |
| HOTAIR | hsa-mir-4514 | 0.817 |
| HOTAIR | hsa-mir-6132 | 0.816 |
| HOTAIR | hsa-mir-4652 | 0.816 |
| HOTAIR | hsa-mir-6888 | 0.816 |
| HOTAIR | hsa-mir-548az | 0.814 |
| HOTAIR | hsa-mir-1237 | 0.813 |
| HOTAIR | hsa-mir-892a | 0.811 |
| HOTAIR | hsa-mir-762 | 0.809 |
| HOTAIR | hsa-mir-1285 | 0.809 |
| HOTAIR | hsa-mir-130b | 0.808 |
| HOTAIR | hsa-mir-6728 | 0.805 |
| HOTAIR | hsa-mir-7112 | 0.805 |
| HOTAIR | hsa-mir-1227 | 0.805 |
| HOTAIR | hsa-mir-130a | 0.803 |
| HOTAIR | hsa-mir-1910 | 0.8 |
| LINC01833 | hsa-mir-335 | 0.999 |
| LINC01833 | hsa-mir-1183 | 0.998 |
| LINC01833 | hsa-mir-1301 | 0.975 |
| LINC01833 | hsa-mir-4778 | 0.974 |
| LINC01833 | hsa-mir-489 | 0.973 |
| LINC01833 | hsa-mir-1253 | 0.952 |
| LINC01833 | hsa-mir-7108 | 0.951 |
| LINC01833 | hsa-mir-5047 | 0.948 |
| LINC01833 | hsa-mir-5089 | 0.937 |
| LINC01833 | hsa-mir-1271 | 0.937 |
| LINC01833 | hsa-mir-128 | 0.937 |
| LINC01833 | hsa-mir-212 | 0.936 |
| LINC01833 | hsa-mir-6844 | 0.936 |
| LINC01833 | hsa-mir-5703 | 0.935 |
| LINC01833 | hsa-mir-4434 | 0.933 |
| LINC01833 | hsa-mir-32 | 0.931 |
| LINC01833 | hsa-mir-4297 | 0.927 |
| LINC01833 | hsa-mir-1299 | 0.925 |
| LINC01833 | hsa-mir-513a | 0.923 |
| LINC01833 | hsa-mir-6827 | 0.919 |
| LINC01833 | hsa-mir-6839 | 0.918 |
| LINC01833 | hsa-mir-216a | 0.918 |
| LINC01833 | hsa-mir-34b | 0.917 |
| LINC01833 | hsa-mir-5088 | 0.916 |
| LINC01833 | hsa-mir-3192 | 0.911 |
| LINC01833 | hsa-mir-6794 | 0.907 |
| LINC01833 | hsa-mir-205 | 0.904 |
| LINC01833 | hsa-mir-548s | 0.903 |
| LINC01833 | hsa-mir-550a | 0.898 |
| LINC01833 | hsa-mir-548p | 0.896 |
| LINC01833 | hsa-mir-4700 | 0.895 |
| LINC01833 | hsa-mir-186 | 0.892 |
| LINC01833 | hsa-mir-27a | 0.89 |
| LINC01833 | hsa-mir-6829 | 0.882 |
| LINC01833 | hsa-mir-3154 | 0.881 |
| LINC01833 | hsa-mir-1260b | 0.88 |
| LINC01833 | hsa-mir-7156 | 0.874 |
| LINC01833 | hsa-mir-27b | 0.868 |
| LINC01833 | hsa-mir-1246 | 0.866 |
| LINC01833 | hsa-mir-498 | 0.865 |
| LINC01833 | hsa-mir-8061 | 0.864 |
| LINC01833 | hsa-mir-765 | 0.863 |
| LINC01833 | hsa-mir-3133 | 0.861 |
| LINC01833 | hsa-mir-4271 | 0.86 |
| LINC01833 | hsa-mir-4516 | 0.859 |
| LINC01833 | hsa-mir-4760 | 0.855 |
| LINC01833 | hsa-mir-6763 | 0.854 |
| LINC01833 | hsa-mir-1276 | 0.851 |
| LINC01833 | hsa-mir-3921 | 0.847 |
| LINC01833 | hsa-mir-513c | 0.847 |
| LINC01833 | hsa-mir-6508 | 0.847 |
| LINC01833 | hsa-mir-580 | 0.846 |
| LINC01833 | hsa-mir-4639 | 0.844 |
| LINC01833 | hsa-mir-1250 | 0.841 |
| LINC01833 | hsa-mir-4776 | 0.835 |
| LINC01833 | hsa-mir-4687 | 0.828 |
| LINC01833 | hsa-mir-1915 | 0.828 |
| LINC01833 | hsa-mir-4653 | 0.827 |
| LINC01833 | hsa-mir-4666b | 0.826 |
| LINC01833 | hsa-mir-1208 | 0.825 |
| LINC01833 | hsa-mir-4509 | 0.824 |
| LINC01833 | hsa-mir-3926 | 0.823 |
| LINC01833 | hsa-mir-4729 | 0.823 |
| LINC01833 | hsa-mir-6799 | 0.82 |
| LINC01833 | hsa-mir-6072 | 0.819 |
| LINC01833 | hsa-mir-6838 | 0.818 |
| LINC01833 | hsa-mir-1290 | 0.817 |
| LINC01833 | hsa-mir-577 | 0.814 |
| LINC01833 | hsa-mir-6894 | 0.806 |
| LINC01833 | hsa-mir-4698 | 0.806 |
| LINC01833 | hsa-mir-6779 | 0.805 |
| LINC01833 | hsa-mir-8070 | 0.805 |
| LINC01833 | hsa-mir-4775 | 0.804 |
| LINC01833 | hsa-mir-3681 | 0.802 |
| LINC01833 | hsa-mir-1305 | 0.801 |
| LINC01833 | hsa-mir-6858 | 0.8 |
| LINC01833 | hsa-mir-4540 | 0.8 |
| BANCR | hsa-mir-4522 | 0.952 |
| BANCR | hsa-mir-143 | 0.896 |
| BANCR | hsa-mir-497 | 0.892 |
| BANCR | hsa-mir-548q | 0.868 |
| BANCR | hsa-mir-424 | 0.845 |
| BANCR | hsa-mir-6788 | 0.821 |
| BANCR | hsa-mir-4646 | 0.814 |
| AC112206.2 | hsa-mir-302f | 0.999 |
| AC112206.2 | hsa-mir-206 | 0.999 |
| AC112206.2 | hsa-mir-1 | 0.998 |
| AC112206.2 | hsa-mir-5004 | 0.993 |
| AC112206.2 | hsa-mir-6764 | 0.992 |
| AC112206.2 | hsa-mir-6824 | 0.991 |
| AC112206.2 | hsa-mir-6781 | 0.987 |
| AC112206.2 | hsa-mir-1299 | 0.986 |
| AC112206.2 | hsa-mir-613 | 0.978 |
| AC112206.2 | hsa-mir-3977 | 0.976 |
| AC112206.2 | hsa-mir-4753 | 0.972 |
| AC112206.2 | hsa-mir-5582 | 0.966 |
| AC112206.2 | hsa-mir-6768 | 0.94 |
| AC112206.2 | hsa-mir-2115 | 0.938 |
| AC112206.2 | hsa-mir-668 | 0.929 |
| AC112206.2 | hsa-mir-421 | 0.926 |
| AC112206.2 | hsa-mir-5001 | 0.925 |
| AC112206.2 | hsa-mir-29b | 0.925 |
| AC112206.2 | hsa-mir-1278 | 0.924 |
| AC112206.2 | hsa-mir-6817 | 0.923 |
| AC112206.2 | hsa-mir-6862 | 0.923 |
| AC112206.2 | hsa-mir-6835 | 0.922 |
| AC112206.2 | hsa-mir-510 | 0.921 |
| AC112206.2 | hsa-mir-5683 | 0.921 |
| AC112206.2 | hsa-mir-520d | 0.917 |
| AC112206.2 | hsa-mir-6844 | 0.917 |
| AC112206.2 | hsa-mir-524 | 0.916 |
| AC112206.2 | hsa-mir-1290 | 0.913 |
| AC112206.2 | hsa-mir-30a | 0.9 |
| AC112206.2 | hsa-mir-3619 | 0.9 |
| AC112206.2 | hsa-mir-2116 | 0.899 |
| AC112206.2 | hsa-mir-4713 | 0.891 |
| AC112206.2 | hsa-mir-4690 | 0.888 |
| AC112206.2 | hsa-mir-6851 | 0.887 |
| AC112206.2 | hsa-mir-4780 | 0.886 |
| AC112206.2 | hsa-mir-30d | 0.885 |
| AC112206.2 | hsa-mir-30e | 0.884 |
| AC112206.2 | hsa-mir-6826 | 0.883 |
| AC112206.2 | hsa-mir-516a | 0.879 |
| AC112206.2 | hsa-mir-516b | 0.879 |
| AC112206.2 | hsa-mir-3191 | 0.878 |
| AC112206.2 | hsa-mir-3152 | 0.878 |
| AC112206.2 | hsa-mir-4742 | 0.876 |
| AC112206.2 | hsa-mir-5699 | 0.874 |
| AC112206.2 | hsa-mir-3116 | 0.874 |
| AC112206.2 | hsa-mir-4731 | 0.872 |
| AC112206.2 | hsa-mir-891a | 0.871 |
| AC112206.2 | hsa-mir-5685 | 0.87 |
| AC112206.2 | hsa-mir-7162 | 0.869 |
| AC112206.2 | hsa-mir-6894 | 0.869 |
| AC112206.2 | hsa-mir-224 | 0.869 |
| AC112206.2 | hsa-mir-6165 | 0.867 |
| AC112206.2 | hsa-mir-7852 | 0.866 |
| AC112206.2 | hsa-mir-4457 | 0.865 |
| AC112206.2 | hsa-mir-7978 | 0.864 |
| AC112206.2 | hsa-mir-597 | 0.863 |
| AC112206.2 | hsa-mir-3156 | 0.86 |
| AC112206.2 | hsa-mir-7154 | 0.858 |
| AC112206.2 | hsa-mir-4303 | 0.857 |
| AC112206.2 | hsa-mir-3925 | 0.855 |
| AC112206.2 | hsa-mir-3685 | 0.854 |
| AC112206.2 | hsa-mir-4665 | 0.854 |
| AC112206.2 | hsa-mir-302c | 0.853 |
| AC112206.2 | hsa-mir-532 | 0.853 |
| AC112206.2 | hsa-mir-629 | 0.851 |
| AC112206.2 | hsa-mir-548b | 0.851 |
| AC112206.2 | hsa-mir-765 | 0.85 |
| AC112206.2 | hsa-mir-152 | 0.849 |
| AC112206.2 | hsa-mir-4709 | 0.849 |
| AC112206.2 | hsa-mir-670 | 0.84 |
| AC112206.2 | hsa-mir-2467 | 0.835 |
| AC112206.2 | hsa-mir-6505 | 0.834 |
| AC112206.2 | hsa-mir-150 | 0.83 |
| AC112206.2 | hsa-mir-330 | 0.83 |
| AC112206.2 | hsa-mir-545 | 0.83 |
| AC112206.2 | hsa-mir-4674 | 0.83 |
| AC112206.2 | hsa-mir-1178 | 0.827 |
| AC112206.2 | hsa-mir-5571 | 0.825 |
| AC112206.2 | hsa-mir-548w | 0.823 |
| AC112206.2 | hsa-mir-548a | 0.823 |
| AC112206.2 | hsa-mir-5581 | 0.823 |
| AC112206.2 | hsa-mir-5706 | 0.822 |
| AC112206.2 | hsa-mir-624 | 0.82 |
| AC112206.2 | hsa-mir-3170 | 0.819 |
| AC112206.2 | hsa-mir-4782 | 0.819 |
| AC112206.2 | hsa-mir-579 | 0.818 |
| AC112206.2 | hsa-mir-9 | 0.815 |
| AC112206.2 | hsa-mir-1234 | 0.814 |
| AC112206.2 | hsa-mir-3670 | 0.811 |
| AC112206.2 | hsa-mir-9 | 0.815 |
| AC112206.2 | hsa-mir-1234 | 0.814 |
| AC112206.2 | hsa-mir-3670 | 0.811 |
| AC112206.2 | hsa-mir-9 | 0.815 |
| AC112206.2 | hsa-mir-7155 | 0.811 |
| AC112206.2 | hsa-mir-3173 | 0.81 |
| AC112206.2 | hsa-mir-3144 | 0.81 |
| AC112206.2 | hsa-mir-4437 | 0.808 |
| AC112206.2 | hsa-mir-3668 | 0.805 |
| AC112206.2 | hsa-mir-548az | 0.805 |
| AC112206.2 | hsa-mir-6124 | 0.804 |
| AC112206.2 | hsa-mir-548as | 0.804 |
| AC112206.2 | hsa-mir-22 | 0.802 |
| AC112206.2 | hsa-mir-548ap | 0.8 |
| AC112206.2 | hsa-mir-6715a | 0.8 |
