# Supplementary material for: Five long non-coding RNAs establish a prognostic nomogram and construct a competing endogenous RNA network in the progression of non-small cell lung cancer
Source: BMC Cancer. 2021 Apr 23;21:457. doi: 10.1186/s12885-021-08207-7 (PMC8067646; doi:10.1186/s12885-021-08207-7)
Supplement: Supplementary file 10 — Additional file 10 : Supplementary Table 4. The qualified lncRNA-miRNA and miRNA-mRNA pairs were constructed in the ceRNA network. [file 12885_2021_8207_MOESM10_ESM.docx]

**Table S4**. The qualified lncRNA-miRNA and miRNA-mRNA pairs were constructed in the ceRNA network.

| Node1 | Node2 | Type |
| --- | --- | --- |
| HOTAIR | hsa-mir-130b-3p | lncRNA |
| AC112206.2 | hsa-mir-1-3p | lncRNA |
| BANCR | hsa-mir-143-5p | lncRNA |
| HOTAIR | hsa-mir-148a-3p | lncRNA |
| HOTAIR | hsa-mir-190a-3p | lncRNA |
| HOTAIR | hsa-mir-204-5p | lncRNA |
| LINC01833 | hsa-mir-205-3p | lncRNA |
| AC112206.2 | hsa-mir-206 | lncRNA |
| FAM83A-AS1 | hsa-mir-218-2-3p | lncRNA |
| AC112206.2 | hsa-mir-30a-3p | lncRNA |
| HOTAIR | hsa-mir-30a-5p | lncRNA |
| HOTAIR | hsa-mir-30c-5p | lncRNA |
| LINC01833 | hsa-mir-34b-3p | lncRNA |
| HOTAIR | hsa-mir-4652-3p | lncRNA |
| FAM83A-AS1 | hsa-mir-516a-3p | lncRNA |
| AC112206.2 | hsa-mir-516a-3p | lncRNA |
| AC112206.2 | hsa-mir-5683 | lncRNA |
| LINC01833 | hsa-mir-577 | lncRNA |
| AC112206.2 | hsa-mir-891a-3p | lncRNA |
| HOTAIR | hsa-mir-93-5p | lncRNA |
| FAM83A-AS1 | hsa-mir-938 | lncRNA |
| AC112206.2 | hsa-mir-9-5p | lncRNA |
| hsa-mir-130b-3p | ANKRD29 | mRNA |
| hsa-mir-130b-3p | ARHGEF26 | mRNA |
| hsa-mir-130b-3p | CEP55 | mRNA |
| hsa-mir-130b-3p | DEPDC1 | mRNA |
| hsa-mir-130b-3p | DLC1 | mRNA |
| hsa-mir-130b-3p | EDN1 | mRNA |
| hsa-mir-130b-3p | EGLN3 | mRNA |
| hsa-mir-130b-3p | FAT3 | mRNA |
| hsa-mir-130b-3p | HEG1 | mRNA |
| hsa-mir-130b-3p | KCNB1 | mRNA |
| hsa-mir-130b-3p | NCKAP5 | mRNA |
| hsa-mir-130b-3p | PPARG | mRNA |
| hsa-mir-130b-3p | SIX4 | mRNA |
| hsa-mir-130b-3p | STARD13 | mRNA |
| hsa-mir-1-3p | ADAM12 | mRNA |
| hsa-mir-1-3p | AGMAT | mRNA |
| hsa-mir-1-3p | ANKRD29 | mRNA |
| hsa-mir-1-3p | C6orf118 | mRNA |
| hsa-mir-1-3p | CBX2 | mRNA |
| hsa-mir-1-3p | EDN1 | mRNA |
| hsa-mir-1-3p | HIF3A | mRNA |
| hsa-mir-1-3p | IL11 | mRNA |
| hsa-mir-1-3p | MME | mRNA |
| hsa-mir-1-3p | MYOCD | mRNA |
| hsa-mir-1-3p | NCAPG | mRNA |
| hsa-mir-1-3p | PPARG | mRNA |
| hsa-mir-1-3p | RCOR2 | mRNA |
| hsa-mir-1-3p | RIMS4 | mRNA |
| hsa-mir-1-3p | RXFP1 | mRNA |
| hsa-mir-1-3p | UHRF1 | mRNA |
| hsa-mir-143-5p | ANKRD29 | mRNA |
| hsa-mir-143-5p | NPNT | mRNA |
| hsa-mir-143-5p | TPPP | mRNA |
| hsa-mir-143-5p | GINS4 | mRNA |
| hsa-mir-148a-3p | CEP55 | mRNA |
| hsa-mir-148a-3p | COLEC12 | mRNA |
| hsa-mir-148a-3p | S1PR1 | mRNA |
| hsa-mir-148a-3p | STARD13 | mRNA |
| hsa-mir-190a-3p | BMP2 | mRNA |
| hsa-mir-190a-3p | C10orf67 | mRNA |
| hsa-mir-190a-3p | GPRIN2 | mRNA |
| hsa-mir-190a-3p | HHIP | mRNA |
| hsa-mir-190a-3p | ID4 | mRNA |
| hsa-mir-190a-3p | IGF2BP1 | mRNA |
| hsa-mir-190a-3p | KCNB1 | mRNA |
| hsa-mir-190a-3p | NEGR1 | mRNA |
| hsa-mir-190a-3p | NTRK3 | mRNA |
| hsa-mir-190a-3p | OTX1 | mRNA |
| hsa-mir-190a-3p | PCDH10 | mRNA |
| hsa-mir-190a-3p | RAB3B | mRNA |
| hsa-mir-190a-3p | SERTM1 | mRNA |
| hsa-mir-190a-3p | SHOX2 | mRNA |
| hsa-mir-190a-3p | SLC24A2 | mRNA |
| hsa-mir-190a-3p | SLC7A11 | mRNA |
| hsa-mir-190a-3p | STARD8 | mRNA |
| hsa-mir-190a-3p | TPPP | mRNA |
| hsa-mir-204-5p | ALPL | mRNA |
| hsa-mir-204-5p | ARHGAP6 | mRNA |
| hsa-mir-204-5p | CHRDL1 | mRNA |
| hsa-mir-204-5p | IL11 | mRNA |
| hsa-mir-204-5p | KIAA1324L | mRNA |
| hsa-mir-204-5p | MYOC | mRNA |
| hsa-mir-204-5p | MYOCD | mRNA |
| hsa-mir-204-5p | MYRF | mRNA |
| hsa-mir-204-5p | PLCXD3 | mRNA |
| hsa-mir-204-5p | SHOX2 | mRNA |
| hsa-mir-204-5p | SIX1 | mRNA |
| hsa-mir-204-5p | SLC1A1 | mRNA |
| hsa-mir-204-5p | SMAD6 | mRNA |
| hsa-mir-204-5p | TPPP | mRNA |
| hsa-mir-204-5p | WWC2 | mRNA |
| hsa-mir-205-3p | HELLS | mRNA |
| hsa-mir-205-3p | SLC7A5 | mRNA |
| hsa-mir-205-3p | STARD8 | mRNA |
| hsa-mir-206 | RIMS4 | mRNA |
| hsa-mir-218-2-3p | SLIT3 | mRNA |
| hsa-mir-30a-3p | COLEC12 | mRNA |
| hsa-mir-30a-3p | KIAA1324L | mRNA |
| hsa-mir-30a-3p | PCSK2 | mRNA |
| hsa-mir-30a-3p | STARD8 | mRNA |
| hsa-mir-30a-5p | CBX2 | mRNA |
| hsa-mir-30a-5p | CELSR3 | mRNA |
| hsa-mir-30a-5p | ERG | mRNA |
| hsa-mir-30a-5p | KIF11 | mRNA |
| hsa-mir-30a-5p | MYBL2 | mRNA |
| hsa-mir-30a-5p | PCDH10 | mRNA |
| hsa-mir-30a-5p | RRM2 | mRNA |
| hsa-mir-30a-5p | SIX4 | mRNA |
| hsa-mir-30a-5p | SLC7A11 | mRNA |
| hsa-mir-30a-5p | SLC7A5 | mRNA |
| hsa-mir-30c-5p | CBX2 | mRNA |
| hsa-mir-30c-5p | CELSR3 | mRNA |
| hsa-mir-30c-5p | DLC1 | mRNA |
| hsa-mir-30c-5p | IL11 | mRNA |
| hsa-mir-30c-5p | KIF11 | mRNA |
| hsa-mir-30c-5p | MYBL2 | mRNA |
| hsa-mir-30c-5p | PCDH10 | mRNA |
| hsa-mir-30c-5p | POLQ | mRNA |
| hsa-mir-30c-5p | RRM2 | mRNA |
| hsa-mir-30c-5p | SIX4 | mRNA |
| hsa-mir-30c-5p | SLC7A5 | mRNA |
| hsa-mir-34b-3p | ACADL | mRNA |
| hsa-mir-4652-3p | GRIK4 | mRNA |
| hsa-mir-4652-3p | MYOCD | mRNA |
| hsa-mir-516a-3p | COL1A1 | mRNA |
| hsa-mir-516a-3p | MYOCD | mRNA |
| hsa-mir-516a-3p | SULF1 | mRNA |
| hsa-mir-5683 | DLC1 | mRNA |
| hsa-mir-5683 | POLQ | mRNA |
| hsa-mir-5683 | SLC7A11 | mRNA |
| hsa-mir-577 | IGF2BP1 | mRNA |
| hsa-mir-891a-3p | IGF2BP1 | mRNA |
| hsa-mir-93-5p | AGMAT | mRNA |
| hsa-mir-93-5p | ANKRD29 | mRNA |
| hsa-mir-93-5p | BMP2 | mRNA |
| hsa-mir-93-5p | CAV1 | mRNA |
| hsa-mir-93-5p | CYBRD1 | mRNA |
| hsa-mir-93-5p | E2F2 | mRNA |
| hsa-mir-93-5p | EGLN3 | mRNA |
| hsa-mir-93-5p | FAM83D | mRNA |
| hsa-mir-93-5p | GINS4 | mRNA |
| hsa-mir-93-5p | HEG1 | mRNA |
| hsa-mir-93-5p | HMGB3 | mRNA |
| hsa-mir-93-5p | KCNB1 | mRNA |
| hsa-mir-93-5p | KIF23 | mRNA |
| hsa-mir-93-5p | KLB | mRNA |
| hsa-mir-93-5p | MELK | mRNA |
| hsa-mir-93-5p | NPNT | mRNA |
| hsa-mir-93-5p | POLQ | mRNA |
| hsa-mir-93-5p | RRM2 | mRNA |
| hsa-mir-93-5p | SLC6A4 | mRNA |
| hsa-mir-93-5p | SLC7A11 | mRNA |
| hsa-mir-93-5p | SMAD6 | mRNA |
| hsa-mir-93-5p | TMEM100 | mRNA |
| hsa-mir-938 | THBS2 | mRNA |
| hsa-mir-9-5p | COLEC12 | mRNA |
| hsa-mir-9-5p | E2F7 | mRNA |
| hsa-mir-9-5p | FERMT1 | mRNA |
| hsa-mir-9-5p | ID4 | mRNA |
| hsa-mir-9-5p | MMP13 | mRNA |
| hsa-mir-9-5p | MSR1 | mRNA |
| hsa-mir-9-5p | NTRK3 | mRNA |
| hsa-mir-9-5p | ONECUT2 | mRNA |
| hsa-mir-9-5p | PRG4 | mRNA |
| hsa-mir-9-5p | RHOV | mRNA |
